# Identification of molecular subtypes based on liquid–liquid phase separation and cross-talk with immunological phenotype in bladder cancer

**DOI:** 10.3389/fimmu.2022.1059568

**Published:** 2022-11-28

**Authors:** Le Sun, Xiao-Ping Liu, Xin Yan, Shaojie Wu, Xiaoyu Tang, Chen Chen, Gang Li, Hankun Hu, Du Wang, Sheng Li

**Affiliations:** ^1^ Department of Urology, Zhongnan Hospital of Wuhan University, Wuhan, China; ^2^ Department of Biological Repositories, Cancer Precision Diagnosis and Treatment and Translational Medicine Hubei Engineering Research Center, Zhongnan Hospital of Wuhan University, Wuhan, China; ^3^ Department of Pharmacy, Zhongnan Hospital of Wuhan University, Wuhan, China; ^4^ The Institute of Technological Sciences, Wuhan University, Wuhan, China

**Keywords:** immunotherapy, cytotoxicity, bladder cancer, liquid-liquid phase separation, molecular subtypes, machine learning, tumor microenvironment

## Abstract

**Background:**

Mounting evidence has demonstrated that an imbalance in liquid–liquid phase separation (LLPS) can induce alteration in the spatiotemporal coordination of biomolecular condensates, which plays a role in carcinogenesis and cachexia. However, the role of LLPS in the occurrence and progression of bladder cancer (BLCA) remains to be elucidated. Identifying the role of LLPS in carcinogenesis may aid in cancer therapeutics.

**Methods:**

A total of 1,351 BLCA samples from six cohorts were retrieved from publicly available databases like The Cancer Genome Atlas, Gene Expression Omnibus, and ArrayExpress. The samples were divided into three distinct clusters, and their multi-dimensional heterogeneities were explored. The LLPS patterns of all patients were determined based on the LLPS-related risk score (LLPSRS), and its multifaceted landscape was depicted and experimentally validated at the multi-omics level. Finally, a cytotoxicity-related and LLPSRS-based classifier was established to predict the patient’s response to immune checkpoint blockade (ICB) treatment.

**Results:**

Three LLPS-related subtypes were identified and validated. The differences in prognosis, tumor microenvironment (TME) features, cancer hallmarks, and certain signatures of the three LLPS-related subtypes were validated. LLPSRS was calculated, which could be used as a prognostic biomarker. A close correlation was observed between clinicopathological features, genomic variations, biological mechanisms, immune infiltration in TME, chemosensitivity, and LLPSRS. Furthermore, our classifier could effectively predict immunotherapy response in patients with BLCA.

**Conclusions:**

Our study identified a novel categorization of BLCA patients based on LLPS. The LLPSRS could predict the prognosis of patients and aid in designing personalized medicine. Further, our binary classifier could effectively predict patients’ sensitivity to immunotherapy.

## Introduction

Bladder cancer (BLCA) is an extremely complex disease, and aberrations occur at the genetic, epigenetic, transcriptomic, epitranscriptomic, proteomic, and phenotypic levels. In a classical view, “hallmarks of cancer” is envisaged to empower cancer malignancy (1). However, various studies showed that intrinsically disordered regions (IDRs) could be the underlying cause of cancer-associated cachexia (2–4). IDRs may undergo liquid–liquid phase separation (LLPS) to form liquid droplets, which affect multiple downstream pathways, including changes in gene expression and histology (5). LLPS is a dynamic process wherein the biomolecular condensates, like various proteins and nucleic acids, turn into liquid aggregates without surrounding membranes (6). A study has shown that LLPS could mediate the spatiotemporal assembly of membraneless organelles, such as stress granules (SGs) and processing bodies (P-bodies) (7).

Various studies have shown that LLPS plays a non-negligible role in various pathological conditions like the occurrence and progression of cancers (5). It has been well established that genetic mutations and transcriptional dysregulation are the underlying cause of cancers. Previous studies have shown that LLPS could induce genetic mutation in cancers (8). For instance, IDRs’ LLPS in NUP98-HOXA9 promotes oncogenes’ activation that induces mutations and carcinogenesis (8). EWS::FLI1, which suppressed nucleolar transcription by LLPS, was a potential target to hinder carcinogenesis (9). Additionally, LLPS plays an important role in regulating multiple pathways associated with cancer, such as DNA damage repair, metabolic rewiring, and immune response (10). Together, these studies indicate the potential role of LLPS in cancers. This would aid in enhancing our understanding of the underlying pathological mechanism of cancers and developing anticancer therapies.

BLCA is the 11th most common cancer worldwide. Approximately 550,000 new cases of BLCA are diagnosed, and 200,000 BLCA-related deaths occur annually (11). Histologically, BLCA cases are categorized into non-muscle-invasive and muscle-invasive (12). Approximately 10% of BLCA cases, characterized by abundant chromosomal alterations and metastasis, would spread beyond the bladder, resulting in a 5-year overall survival (OS) rate of only 5% to 30% (13). A comprehensive genetic analysis performed by The Cancer Genome Atlas (TCGA) revealed subtypes closer to native biological BLCA, confirming that the pathogenesis of BLCA is more complex than the previous understanding (12). Transcriptomic signatures of patients with BLCA have been identified and used to construct models that can predict the prognosis and response to immune checkpoint blockade (ICB) in BLCA patients (14). However, the performance of most prognostic models was not satisfactory in clinical settings. Meanwhile, the only four targeted drugs available nowadays harbored limited scope of application in BLCA (15). Further, radiotherapy induces an immunosuppressive tumor microenvironment (TME), which leads to cancer recurrence (16, 17). Therefore, more personalized and effective biomarkers are required for BLCA cases. Cachexia in patients with BLCA is caused by several LLPS-related factors; however, previous studies have only analyzed the association between single molecules associated with LLPS and cancers rather than exploring the interaction between multiple LLPS-related genes in cancers (8, 9). Therefore, it is necessary to study the correlation between LLPS-related genes and heterogeneities in TME to analyze LLPS patterns in BLCA. To address these concerns, in this study, we have identified LLPS-relevant subtypes and evaluated LLPS-related genes by analyzing data from 1,351 patients with BLCA cases.

## Materials and methods

### Data sources and process

**Figure 1:**
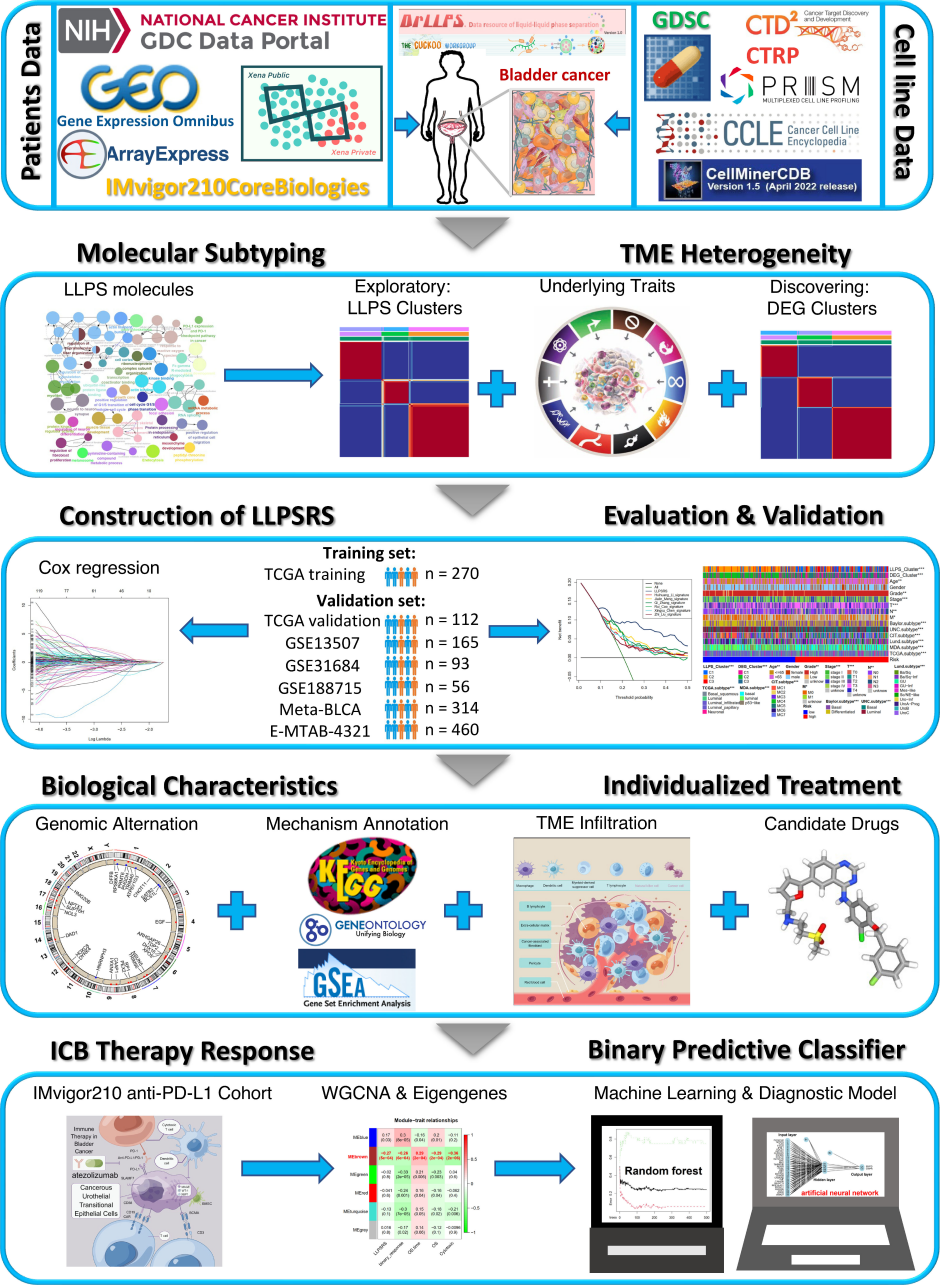
Overview of the flow diagram for this study.

The overall workflow of our study is shown in **Figure 1**. The data sources and workflow details are shown in the **Supplementary Material**.

### Identification of liquid–liquid phase separation-related subtypes

Based on several LLPS-related genes, the patients with BLCA were classified into different subtypes using non-negative matrix factorization (NMF). The patients were classified into three LLPS clusters: C1, C2, and C3. The biological and clinicopathological features of the three subtypes were investigated. The workflow is summarized in the **Supplementary Material**.

### Construction and evaluation of liquid–liquid phase separation-related risk score

Based on previous studies, the individual LLPS patterns were identified. Stepwise multivariate Cox regression analysis was performed to create a scoring system called LLPS-related risk score (LLPSRS) (18). The LLPSRS formula is as follows:


$$\text{LLPSRS}=\sum_{i=1}^{n}Coef_{i}\times {\left(LLPS\;genes\right)}_{i}$$

The performance of the LLPSRS formula was further evaluated to predict the clinical outcomes of BLCA patients. The robustness and versatility of the LLPSRS formula were also validated. The details of the methodology are described in the **Supplementary Material**.

### Establishment of an artificial neural network

An artificial neural network (ANN) was established using a binary classifier to identify patients who might benefit from ICB. The formula for calculating classification score using the ANN model is as follows:


$$\text{neuraHF}=\sum_{i=1}^{n}{\left(\text{Neural Network Weight}\right)}_{i}\times {\left(\text{Gene Expression}\right)}_{i}$$

The details of the procedure are described in the **Supplementary Material**.

### Statistical analysis

All statistical analyses were conducted using R (https://www.r-project.org/). The Wilcoxon test was used to compare two groups, and the Kruskal–Wallis test was used to compare more than two groups. The statistical details and experimental methods are summarized in the **Supplementary Material**.

## Results

### Identification of liquid–liquid phase separation-related molecular subtypes in bladder cancer

**Figure 2:**
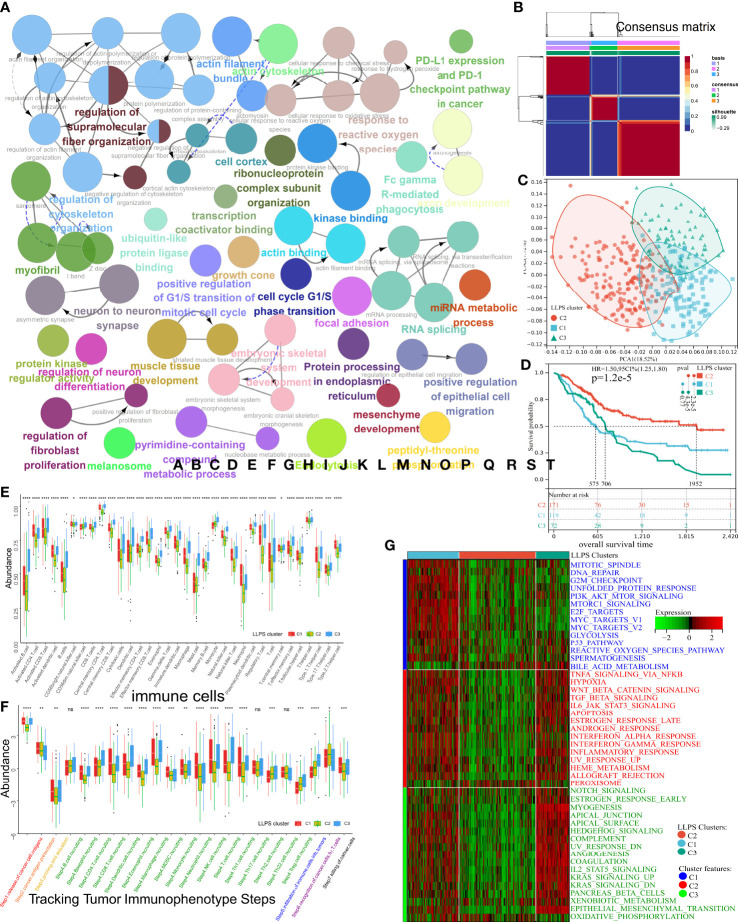
Subtype identification, TME infiltration characteristics, and biological signal features of three distinct LLPS clusters in BLCA. **(A)** The GO terms and KEGG pathway enrichment analysis of 586 prognostic LLPS-related genes. The different colors represent different terms or pathways. **(B)** Consensus map of NMF based on 586 prognostic LLPS-related genes when k = 3. **(C)** PCA plot to distinguish LLPS clusters. **(D)** KM curve exhibited significantly different OS among LLPS clusters in TCGA-BLCA cohort. **(E, F)** Differences in 34 TME-infiltrating cells and steps of the cancer immunity cycle among LLPS clusters. **(G)** Heatmap illustrated cancer hallmarks’ enrichment among three LLPS clusters. Red represents high scores, and green represents low scores. The line in the box represents the median value, and the asterisks represent the p-value (*p< 0.05; **p< 0.01; ***p< 0.001; ****p< 0.0001); the statistical analyses were performed by the Kruskal–Wallis test. TME, tumor microenvironment; LLPS, liquid–liquid phase separation; BLCA, bladder cancer; GO, Gene Ontology; KEGG, Kyoto Encyclopedia of Genes and Genomes; NMF, non-negative matrix factorization; PCA, principal component analysis; KM, Kaplan–Meier; OS, overall survival. ns, no significance.

**Figure 1** shows a flow diagram that systematically describes our study. A total of 3,633 LLPS-related genes were identified from TCGA-BLCA cohort and extracted from the data resource of LLPS (DrLLPS) (19) of which a total of 586 prognostic genes were identified using univariate Cox regression analysis (p< 0.01). To determine the impact of these genes on BLCA, the Gene Ontology (GO) and Kyoto Encyclopedia of Genes and Genomes (KEGG) pathway enrichment analyses were performed. These genes enriched processes associated with the extracellular matrix, immunoreaction, transcription, proliferation, and metabolism (**Figure 2A**). The patients from TCGA-BLCA cohort were categorized into three LLPS clusters based on the expression of 586 genes using NMF (**Supplementary Figures 1A, B**, **Figure 2B**). Principal component analysis (PCA) was further used to validate the differential expression of 586 genes in three clusters, and the clusters’ similar consistency could be distinguished (**Figure 2C**). The Kaplan–Meier (KM) survival curve revealed significant differences in the prognoses of patients among three clusters (log-rank test, p< 0.0001). The clinical outcomes of patients in C2 were significantly better compared to those in C1 and C3 (**Figure 2D**, **Supplementary Table 2**). To determine the reproducibility of LLPS clusters, three external BLCA cohorts were integrated into a meta-BLCA cohort, and three distinct clusters were identified as anticipated (**Supplementary Figures 1C–E**). A significant difference was observed in the prognoses of patients among the three clusters (p< 0.001); the prognoses of patients in C2 were the best, thereby confirming that three robust LLPS clusters exist in BLCA (**Supplementary Figure 1F**).

### Identification of tumor microenvironment characteristics and biological features in liquid–liquid phase separation clusters

Various studies have demonstrated LLPS’s correlation with dysregulation in the TME remodeling and signaling pathways. Hence, the characteristics of TME were analyzed in three LLPS clusters. A decrease in levels of most tumor-infiltrating immune cells (TIICs) like CD4^+^T, CD8^+^T, NK, dendritic cells, and macrophages was observed in cluster C2 (**Figure 2E**). In tracking tumor immunophenotype (TIP), a significant increase in antitumor immune responses was observed in C1 and C3 compared to C2; however, these results were inconsistent with survival outcomes (**Figure 2F**). Next, the representative hallmark gene sets of the three clusters were visualized. The hallmarks of C1 enriched oncogenic signaling pathways like the PI3K-AKT-mTOR, P53, mTORC1, and MYC signaling pathways. The hallmarks of C3 enriched the stromal signatures like epithelial–mesenchymal transition (EMT) and angiogenesis. However, the downregulation of hallmarks associated with immune responses like IL6-JAK-STAT3 or NF-κB-TNFα signaling pathways was observed in C2 (**Figure 2G**). A previous study has shown that TME can be divided into three immunophenotypes: inflamed, excluded, and desert (20). Despite the abundance of TIICs in excluded TME, the TIICs only surrounded the parenchyma. No TIICs were observed in the parenchyma, which indicates that the stromal barrier surrounding the tumor inhibited the cytotoxic effects of TIICs in the TME. Since interstitial activation could suppress the effect of TIICs, the differential enrichment of 12 BLCA signatures (21) was explored among three LLPS clusters (**Supplementary Figure 1G**). As expected, C1 was of basal subtype featured by heightened IFN response, mitochondrial dysfunction, etc., which indicates damage caused by inflammation and cancer development. C3 was featured by the presence of EMT, etc., indicating fibrosis and muscle invasion. Meanwhile, cluster C2 was of a luminal subtype with Ta stage and high differentiation grade; low infiltration of TIIC could likely be due to small tumor size. Further, the enrichment of processes associated with stromal activation, mismatch repair, and immune response-relevant (SA-MR-IR) signatures was determined in the three LLPS clusters (**Supplementary Figure 1H**). The processes enriched in patients in C1 were mismatch repair, including homologous recombination, base excision, and repair. C3 was characterized by stromal activation, including angiogenesis, Pan-F-TBRS, and EMT. Downregulation in processes related to an immune response, like CD8^+^T effector and immune checkpoint, was observed in cluster C2. These results suggested that the TME of three LLPS clusters had distinct immunophenotypes and enriched different oncogenic processes. Together, these results indicated that LLPS played an indispensable role in BLCA. Further, 19 oncogenic pathways’ enrichment was analyzed among LLPS clusters (**Supplementary Figure 1I**) (14). As expected, the pathways enriched in C1 were associated with the cell cycle, including activated MYC and PI3K signaling pathways. The WNT signaling pathway was enriched in C3, confirming the increased EMT, metastasis, and muscle invasion. Interestingly, the Hippo, NOTCH, and RAS pathways were inhibited in C2. Thus, the unique TME characteristics of three LLPS clusters were analyzed.

### Comprehensive analysis of differentially expressed genes among liquid–liquid phase separation clusters of bladder cancer

**Figure 3:**
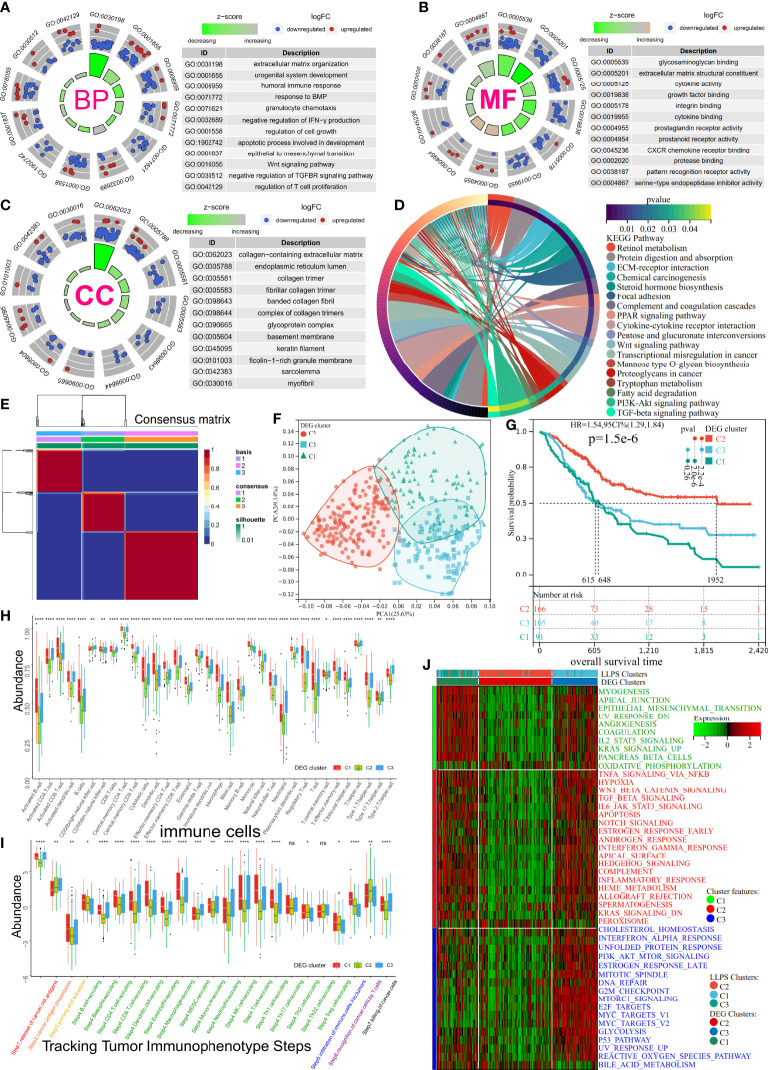
Subtype identification, TME infiltration characteristics, and biological signal features of three distinct DEG clusters in BLCA. **(A–C)** The GO terms enrichment analysis (BP, Biological Process; CC, Cellular Component; MF, Molecular Function) of 470 DEGs. **(D)** The KEGG pathway enrichment analysis of 470 DEGs. **(E)** Consensus map of NMF based on 197 prognostic LLPS-related DEGs when k = 3. **(F)** PCA plot for 197 prognostic LLPS-related DEGs’ expression. **(G)** KM curve exhibited significantly different OS among DEG clusters in TCGA-BLCA cohort. **(H, I)** Differences in 34 TME-infiltrating cells and steps of the cancer immunity cycle among DEG clusters. **(J)** Heatmap illustrating cancer hallmarks’ enrichment among three DEG clusters. Red represents high scores, and green represents low scores. The line in the box represents the median value, and the asterisks represent the p-value (*p< 0.05; **p< 0.01; ***p< 0.001; ****p< 0.0001); the statistical analyses were performed by the Kruskal–Wallis test. TME, tumor microenvironment; DEG, differentially expressed gene; BLCA, bladder cancer; GO, Gene Ontology; KEGG, Kyoto Encyclopedia of Genes and Genomes; NMF, non-negative matrix factorization; LLPS, liquid–liquid phase separation; KM, Kaplan–Meier. ns, no significance.

To unravel the potential biological behavior of three LLPS clusters, a total of 470 differentially expressed genes (DEGs) were identified and annotated (**Supplementary Figure 2A**). These DEGs significantly enriched the pathways associated with metabolism reprogramming, dyssecretosis, increase in cell-autonomous proliferation, alteration biosynthesis, extracellular matrix, and immunoediting (**Figures 3A–D**). To explore a more accurate classification of BLCA subtypes and uncover underlying mechanisms, TCGA-BLCA cohort was classified into three clusters based on differential expression of 197 prognosis-related genes (p< 0.01) using NMF (**Supplementary Figures 2B, C**, **Figure 3E**). The patients were classified into three clusters: DEG-C1, C2, and C3. A total of 99 patients with BLCA were classified in C1, 175 patients with BLCA were grouped in C2, and 108 patients with BLCA were classified in C3 (**Figure 3E**). PCA was used to validate the expression patterns of 197 DEGs in three DEG clusters, and similar consistency was distinguished (**Figure 3F**). Additionally, the KM curve showed significant differences in the prognoses of patients among three DEG clusters (log-rank test, p< 0.0001). The OS of patients in DEG-C2 was significantly better compared to that of patients in DEG-C1 and C3 (**Figure 3G**). Most patients in LLPS-C2 were classified in DEG-C2 (166/181 = 91.72%), 81.30% of patients in LLPS-C1 (100/123) were reassigned to DEG-C3, and 87.18% of patients in LLPS-C3 (68/78) were included in DEG-C1. Eventually, the TME characteristics of three DEG clusters were analyzed, and the results were similar to our previous results (**Figures 3H–J**, **Supplementary Figures 2D–F**, **Supplementary Table 3**).

### Establishment and evaluation of liquid–liquid phase separation-related risk score

**Figure 4:**
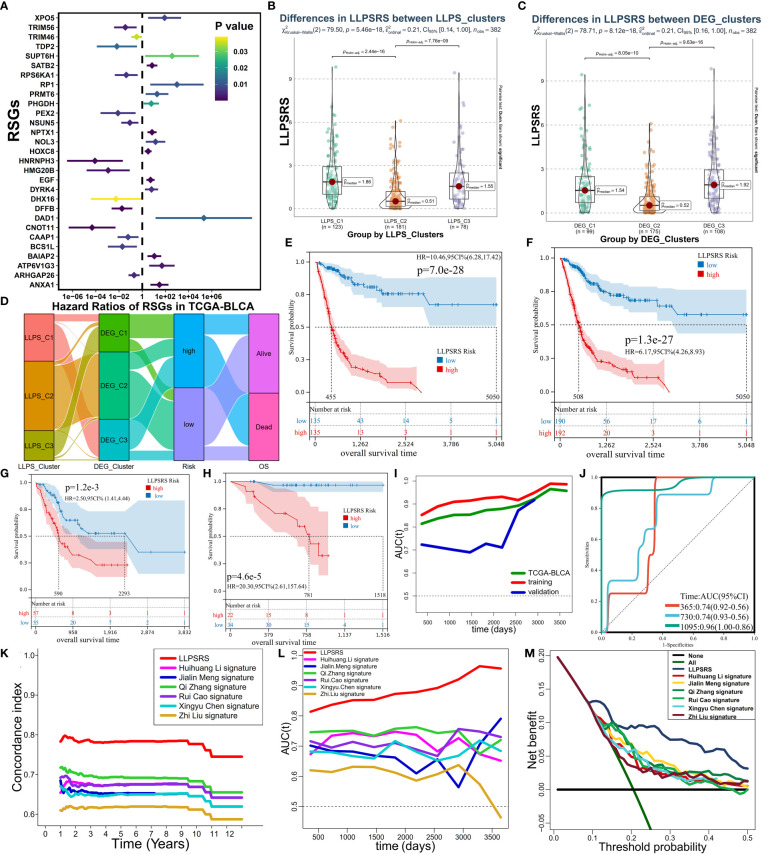
Establishment and evaluation of LLPSRS. **(A)** Forest plot of hazard ratios with 95% CI for 29 RSGs by univariate Cox. **(B)** Sankey diagram showing the changes of LLPS, DEG clusters, risk, and OS in TCGA-BLCA cohort. **(C, D)** Comparison of LLPSRS among LLPS and DEG clusters. **(E–H)** KM curves of LLPSRS in the training, TCGA-BLCA, validation, and GSE188715 cohorts. **(I)** Time-dependent ROC–AUC values plotted for different durations of survival for LLPSRS in the training, TCGA-BLCA, and validation sets. **(J)** ROC curves of LLPSRS in GSE188715 cohort. **(K–M)** Time-dependent C-index, ROC–AUC, and DCA showing a measure of LLPSRS with six prognostic signatures with the survival of patients in TCGA-BLCA cohort. LLPSRS, liquid–liquid phase separation-related risk score; DEG, differentially expressed gene; OS, overall survival; LLPS, liquid–liquid phase separation; KM, Kaplan–Meier; ROC, receiver operating characteristic; AUC, area under the receiver operating characteristic curve; DCA, decision curve analysis.

Given the heterogeneity and complexity of LLPS, the LLPSRS was calculated to quantify LLPS-related clusters and predict patients’ prognoses. In the training cohort, 424 prognosis-related genes were identified. Univariate and least absolute shrinkage and selection operator Cox regression analyses identified 60 genes as candidate genes (**Supplementary Figures 3A, B**). Next, stepwise multivariate Cox proportional regression analysis was used to screen for 29 robust genes to calculate LLPSRS (**Figure 4A**, **Supplementary Table 4**). In the training cohort, patients were classified into the high- (n = 135) and low-risk (n = 135) subgroups based on median LLPSRS as a cutoff value. The patients in the validation cohort were also divided based on these criteria. The difference in the distribution of subtypes, risk, and OS was calculated, and the results revealed significant differences in LLPSRS among LLPS or DEG clusters (p< 0.0001, **Figures 4B–D**). The LLPSRS of patients in C2 was lower compared to that of patients in C1 and C3, thus suggesting that LLPSRS may be useful in predicting BLCA subtypes.

**Figure 5:**
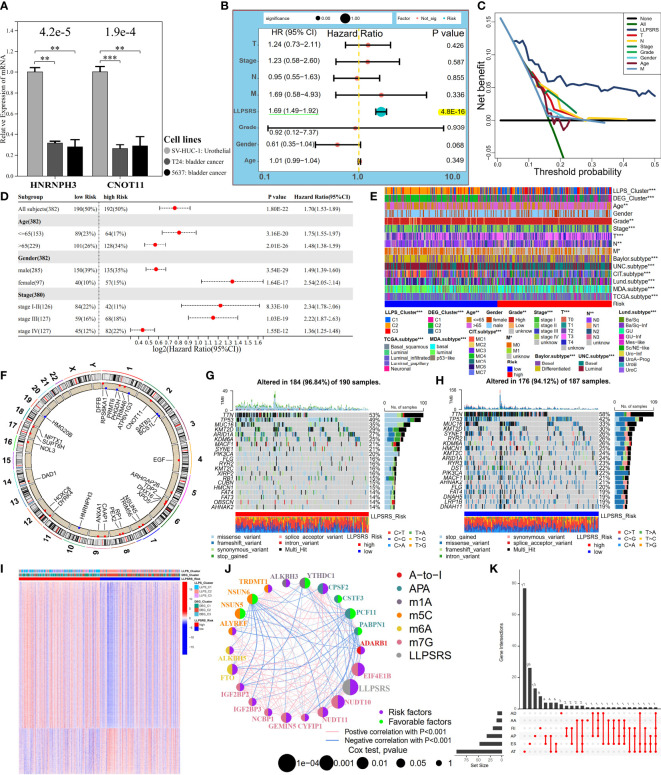
LLPSRS’s association with clinicopathological features, genomic variations, and epigenetic alternations. **(A)** Differential transcript levels of HNRNPH3 and CNOT11 in BLCA and normal urothelial cell lines. **(B, C)** Multivariate Cox analysis and DCA of LLPSRS and seven clinical traits in TCGA-BLCA cohort. **(D)** Univariate Cox analysis of LLPSRS with OS in several stratifications. **(E)** Differential clinical traits and molecular subtypes between the high- and low-risk subgroups (chi-square test). **(F)** Twenty-nine RSGs’ location of CNV alterations on chromosomes. **(G, H)** Waterfall plots showing the mutation distribution of top 20 mutated genes in two subgroups. **(I)** Heatmap of DMCGs in two subgroups. **(J)** The interactive network and prognostic implications of LLPSRS and 21 RNA editing regulators. The left half-circle represents different RNA modification patterns, and the right half-circle represents whether these regulators were risk or protective factors for OS. The color of lines indicates correlations between abovementioned regulators, and the size of circles represents p-values for prognostic implications of these regulators. **(K)** Statistics on the counts and types of 161 DEGs with AS. The asterisks represent the p-value (*p< 0.05; **p< 0.01; ***p< 0.001). LLPSRS, liquid–liquid phase separation-related risk score; BLCA, bladder cancer; DCA, decision curve analysis; OS, overall survival; CNV, copy number variation; DMCGs, differentially methylated CpG islands; AS, alternative splicing.

Subsequently, the ability of LLPSRS to predict the prognosis was determined. In training, TCGA-BLCA, validation, and several external cohorts, the prognoses of patients with high LLPSRS were poor (**Figures 4E–H**, **Supplementary Figure 3F–I**). The area under the receiver operating characteristic (ROC) curve (AUC) was used to validate the performance of LLPSRS to predict the OS of patients with BLCA (**Figures 4I, J**). In TCGA-BLCA cohort and training and validation cohorts, a decrease in the expression of 14 genes that conferred protection was observed, while 15 risk-associated genes were upregulated as LLPSRS increased (**Supplementary Figures 3C–E**). In summary, these results determined the utility and robustness of LLPSRS in predicting the clinical outcomes of patients with BLCA. In other publicly available cohorts, most patients with BLCA were Caucasians or Africans, whereas patients with BLCA in GSE188715 were Chinese; therefore, LLPSRS could be used to predict patients’ prognoses from different ethnicities. Moreover, compared to the performance of the previously published six prognostic models (14, 18, 22–25), the performance of our model was better on several appraisal algorithms. In TCGA-BLCA cohort, our model showed the highest net benefit, concordance index, and AUC, thus confirming that the adaptability of our model was better compared to that of the previously published six models (**Figures 4K–M**, **Supplementary Figure 3J**). Since our retrospective study was currently restricted to retrospective studies, the differential expression of seven core genes from 29 LLPSRS-related genes (RSGs) was verified *in vitro* (**Figure 5A**, **Supplementary Figure 4A**). qRT-PCR was used to study the differential expression of these genes between one bladder and two BLCA cell lines. The results revealed a significant increase in the expression of five genes in BLCA cell lines, consistent with results *via* TCGA-BLCA cohort; however, no difference in the expression of the other two genes was observed.

Since LLPSRS was related to the advanced stage of cancer, the performance of LLPSRS was compared with clinicopathological features in predicting patients’ OS. The univariate and multivariate Cox regression analyses were performed on TCGA-BLCA cohort, and the results showed that the hazard ratios of LLPSRS were 1.70 and 1.69 (p< 0.0001), respectively. This indicates that the performance of LLPSRS to predict prognosis was robust (**Figure 5B**, **Supplementary Figure 4B**). Moreover, the AUC and decision curve analysis (DCA) results confirmed the highest efficacy of LLPSRS over other clinical parameters (**Figure 5C**, **Supplementary Figure 4C–G**). Furthermore, to compensate for bias caused by differences in clinicopathological features, univariate Cox analysis was performed on subgroups with different clinicopathological features. It demonstrated that LLPSRS was an independent prognostic factor after adjustment (**Figure 5D**). The molecular subtypes aid in designing personalized treatment (26). Further, an association between several BLCA subtypes (21) and LLPSRS exists. Hence, the differences in the clinical landscape between the two subgroups in TCGA-BLCA cohort were explored (**Figure 5E**). The patients in the high-risk subgroup had a basal subtype characterized by high malignancy, whereas the patients in the low-risk subgroup had a more differentiated luminal subtype, and their clinical outcomes were better (chi-squared test, p< 0.05). Furthermore, the clinicopathological features of patients in the low-risk subgroup were similar to those in C2. Therefore, these results indicated that the LLPSRS could be used as a biomarker for predicting heterogeneity and designing personalized therapy for patients with BLCA.

### Depicting the landscape of genomic variations and epigenetic alternations

To determine the underlying genomic alterations caused by LLPSRS, the genomic variations between the two subgroups were explored. The copy number variations (CNVs) in RSGs on chromosomes were analyzed and visualized. The results showed significant amplifications in 16 RSGs, whereas five RSGs harbored deletions (**Figure 5F**, **Supplementary Figure 4H**). Furthermore, the mutation landscape of the top 20 mutated RSGs is shown in **Figures 5G, H**. The patients in the high-risk subgroup had distinct mutation patterns, the top three mutated genes were observed in patients from the same subgroup, and there was a difference in the abundance of other mutated genes in the patients from the two subgroups. Moreover, as potential indices for ICB response and neoantigens epitopes, tumor mutational burden (TMB) and purity data were obtained. A negative correlation was observed between TMB, tumor purity, and the LLPSRS (**Supplementary Figures 4I, J**, p< 0.05). In addition, the interconnection and mutation landscape of RSGs were visualized (**Supplementary Figures 4K, L**). In addition to genomic mutations, epigenetic aberrations play an important role in oncogenesis. Changes in DNA methylation pattern, which plays an important role in pre-transcriptional modification, were next explored. Previous studies have shown that the prognoses of patients with DNA hypomethylation were poor, whereas patients with DNA hypermethylation may experience cachexia (27, 28). A total of 26,583 differentially methylated CpG islands (DMCGs) were identified (**Figure 5I**). The methylation levels in patients in the high-risk subgroup were higher, which confirms our hypothesis. Furthermore, 240 genes were identified as DNA methylation driver genes (MET-DGs). These genes enriched pathways associated with biosynthesis disorders, cancer, and cell proliferation pathways, which indicates that these genes are involved in activating various oncogenic pathways (**Supplementary Figure 5A**). The top 35 MET-DGs were visualized, and the results revealed that the LLPSRS was characterized by the hypermethylation of tumor suppressor genes (**Supplementary Figure 5B**).

Of these 29 RSGs, NSUN5 is an RNA methyltransferase responsible for 5-methylcytidine (m^5^C) modification, whereas DHX16 and HNRNPH3 regulate alternative splicing (AS) during pre-mRNA splicing. Knockdown of NSUN5 expression reduces cell proliferation (29), DHX16 is a biomarker for immune-related adverse events (irAEs) (30), and HNRNPH3 directly alters the mRNA splicing of proto-oncogene MST1R (31). Since RNA editing and AS are key regulators of carcinogenesis, the correlation between the LLPSRS and post-transcriptional regulation was explored. The expression of 95 RNA-editing regulators in different subgroups was analyzed, and the results revealed differential expression in most RNA-editing regulators between two subgroups (**Supplementary Figures 5C–E**). Next, a correlation was observed between 786 out of 1,416 DEGs and 71 RNA-editing regulators. These genes were annotated as hormone secretion, cell division, metabolic rewiring, and immunosuppressive TME (|R| > 0.3, p< 0.0001, **Supplementary Figures 5F, G**). A network was constructed using 21 RNA-editing regulators and LLPSRS to demonstrate their correlation and prognostic roles. Interestingly, a correlation was observed between LLPSRS and most regulators (**Figure 5J**, univariate Cox regression analysis, p< 0.05). In addition to the correlation between LLPSRS and m^5^C regulators, a correlation between LLPSRS and other regulators was also observed. This indicates that diverse crosstalk between LLPSRS and RNA-editing regulators plays a crucial role at the epitranscriptomic level in BLCA. Moreover, 483 AS events were observed in 161 DEGs. These genes enriched pathways associated with carcinogenesis and immunogenicity, thus implying that LLPSRS may regulate these pathways by AS of DEG (**Figure 5K**, **Supplementary Figure 5H**). Correlation networks were constructed between 37 AS events and 22 splicing factors (**Supplementary Figure 5I**). Taken together, our results demonstrated that the LLPS-mediated epigenetic alterations played an important role in the progression of BLCA.

### Guidance of liquid–liquid phase separation-related risk score on potentially relevant biological mechanisms

**Figure 6:**
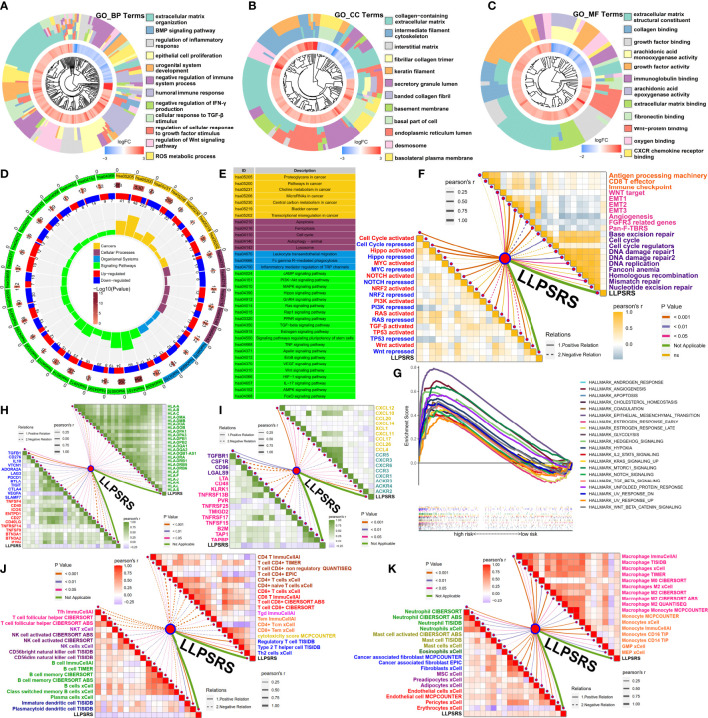
Guidance of LLPSRS on potential biological mechanisms and immunoediting. **(A–C)** DEGs’ annotation of GO terms (BP, Biological Process; CC, Cellular Component; MF, Molecular Function). **(D, E)** DEGs’ annotation of KEGG pathways. **(F)** LLPSRS’s correlations with SA-MR-IR signatures (immune response, orange; stromal activation, pink; mismatch repair, purple) and oncogene pathways (activated, red; repressed, blue). **(G)** GSEA of hallmark gene sets. **(H)** LLPSRS’s correlations with MHC molecules (green) and immune checkpoints (inhibitory, blue; stimulatory, red). **(I)** LLPSRS’s correlations with immunomodulators (immune inhibitor, purple; immunostimulator, magenta), chemokines (golden), and their receptors (cyan). **(J)** LLPSRS’s correlations with lymphocytes (CD4^+^T, brown; CD8^+^T, red; γδT, pink; Tm, orange; cytotoxicity, golden; Th2 and Treg, blue; Tfh, magenta; NKT, indigo; NK, purple; B cell, green; DC, dark blue). **(K)** LLPSRS’s correlations with mononuclear (macrophage, magenta; monocyte, orange) and stromal cells (neutrophil, green; mast cell, yellow green; eosinophil, dark green; CAF, blue; mesenchymal cell, purple; vascular cell, red). LLPSRS, liquid–liquid phase separation-related risk score; DEG, differentially expressed gene; GO, Gene Ontology; KEGG, Kyoto Encyclopedia of Genes and Genomes; GSEA, gene set enrichment analysis.

The GO and KEGG pathway enrichment analyses were performed using DEGs to decipher LLPSRS-relevant biological mechanisms using KOBAS-i (32). The correlation between the LLPSRS, SA-MR-IR, and oncogene signatures was further explored. The LLPSRS was associated with metabolic rewiring, cell-autonomous hyperproliferation, immunoediting, and biosynthesis derangements, thus indicating that LLPSRS played an important role in carcinogenesis (**Figures 6A–E**). Higher LLPSRS reveals persistent stromal activation and transcriptional dysregulation, thus implying tumor cell-autonomous proliferation and excluded immunophenotype (**Figure 6F**). Meanwhile, a positive correlation was observed between LLPSRS and a decrease in the expression of tumor suppressor genes, as well as an increase in oncogenes’ expression like PI3K, RAS, and TGF-β, thereby suggesting a higher degree of malignancy. As expected, the gene set enrichment analysis results revealed that hallmarks of cancers like angiogenesis, EMT, and the TGF-β and WNT signaling pathways were upregulated in the patients in the high-risk subgroup. This indicates the frequent occurrence of cachexia-relevant signatures and an increase in the activation of oncogenes (**Figure 6G**).

### Correlation between liquid–liquid phase separation-related risk score and intrinsic as well as extrinsic immunoediting

As discussed previously, LLPSRS-related mechanisms play an important role in regulating immune responses; hence, the correlation between LLPSRS and intrinsic as well as extrinsic immunoediting was analyzed. A negative correlation was observed between LLPSRS and MHC receptors. A positive correlation was observed between LLPSRS and immunosuppressive checkpoints, thus indicating an increase in immune evasion by tumor cells and a decrease in immunogenicity. Interestingly, a correlation was observed between low LLPSRS and some ICB-related genes like (CTLA4, LAG3, PDCD1, etc.), thus indicating higher sensitivity of patients to ICB (**Figure 6H**). Apart from the chemokines and their receptors, a positive correlation was observed between the protooncogenes and LLPSRS. A negative correlation was observed between other tumor suppressors and LLPSRS. On the contrary, a negative correlation was observed between several immune inhibitors, immune stimulators, and LLPSRS (**Figure 6I**). Considering the multiple complex roles of immunomodulators, the association between immunomodulators and LLPSRS was insufficient to elucidate overall immunological features. The extrinsic immunoediting indicated by TIICs was equally important since they revealed a functional repertoire of antitumor immunity. A correlation between the LLPSRS and TIICs was observed. Increased levels of antitumor TIICs, like CD4^+^T and CD8^+^T, were observed, whereas the levels of pro-tumor TIICs like Treg and Th2 were low in patients in the low-risk subgroup (**Figure 6J**). As expected, a positive correlation was observed between myeloid cells, stromal cells, and LLPSRS, thereby indicating an increase in damage caused by inflammation and interstitial activation (**Figure 6K**). Together, these results suggested that LLPSRS played an important role in immunoediting, which indicated that patients with lower LLPSRS had higher immunogenicity and sensitivity to ICB.

### Liquid–liquid phase separation-related risk score was a promising biomarker for predicting the efficacy of adjuvant treatments

**Figure 7:**
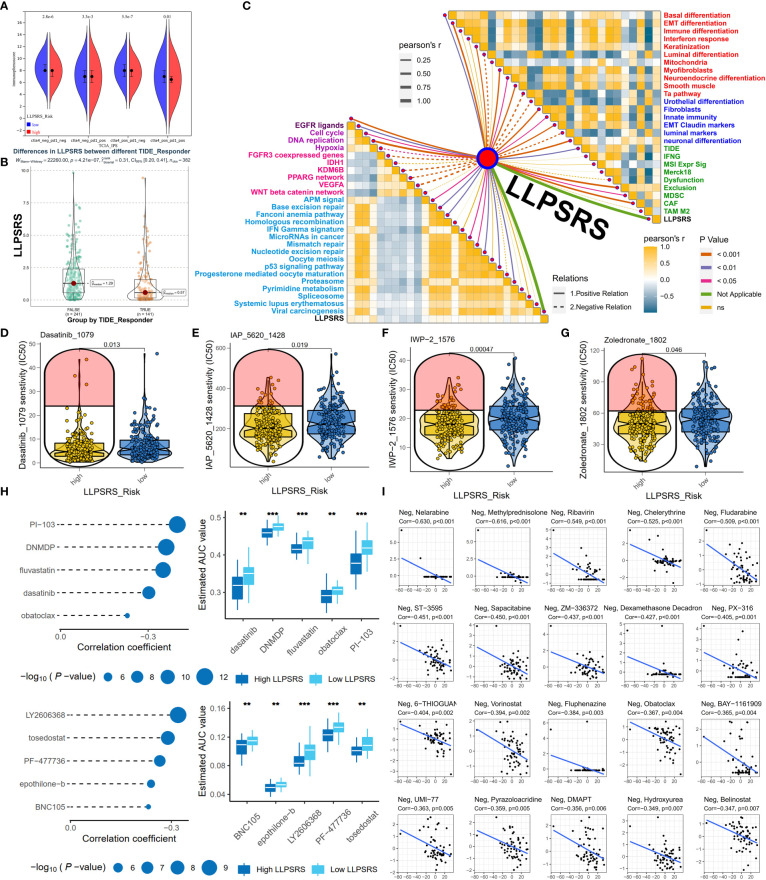
LLPSRS might be a promising biomarker to predict the efficacy of adjuvant treatments. **(A)** Comparison of immunophenoscore between two subgroups. **(B)** Comparison of LLPSRS between responders and non-responders predicted by TIDE. **(C)** LLPSRS’s correlations with therapeutic signatures (EGFR ligands, deep purple; radiotherapy, purple; chemotherapy, magenta; irAEs, sky blue), 12 BLCA signatures (red), tumor progression biomarkers (blue), and additional output of TIDE platform (green). **(D–G)** Dasatinib, IAP_5620, IWP-2, and zoledronate showed lower IC50 value in high-risk subgroups *via* GDSC. **(H)** Five CTRP-related and five PRISM-related compounds were identified by correlation between LLPSRS and AUC value. **(I)** Twenty CellMiner-related potential drugs for patients with higher LLPSRS were identified by correlation between LLPSRS and G150 value. The line in the box represents the median value, and the asterisks represent the p-value (**p< 0.01; ***p< 0.001); the statistical analyses were performed by the Mann–Whitney and Spearman’s correlation test. LLPSRS, liquid–liquid phase separation-related risk score; irAEs, immune-related adverse events; BLCA, bladder cancer; AUC, area under the receiver operating characteristic curve.

The correlation between LLPSRS and anticancer drug regimens was next determined, which could aid in designing precision medicine at the pharmacogenomics level. First, the immunophenoscore of patients in the low-risk group was high, regardless of the status of indicators, which suggests better efficacy of ICB (**Figure 7A**). As expected, the Tumor Immune Dysfunction and Exclusion analysis revealed that the patients who responded to ICB treatment had higher LLPSRS. This indicates that LLPSRS could predict the efficacy of ICB treatment (**Figure 7B**). Furthermore, the association between LLPSRS and gene signatures of adjuvant treatments was explored (**Figure 7C**). A negative correlation was observed between LLPSRS and oncogenic pathways, and a positive correlation was observed between the predicted EGFR pathway, radiotherapy, and irAEs. Therefore, patients with higher LLPSRS were sensitive to EGFR-targeted therapies and radiotherapy, whereas the patients with low LLPSRS were sensitive to oncogenes’ blockades. Additionally, an inverse correlation was observed between LLPSRS and Ta stage, luminal, and urothelial differentiation. The patients in the high-risk subgroup were characterized by immune evasion and dysfunction, and neuronal and basal differentiation, thereby indicating that patients in the high-risk group could benefit from neoadjuvant chemotherapy, whereas ICB might induce hyperprogression.

Although adjuvant treatment was a major breakthrough in cancer therapeutics, chemotherapy is still an indispensable part of BLCA treatment. To determine the use of LLPSRS in determining BLCA treatment, data on experimentally or clinically used drugs in BLCA were retrieved from the Genomics of Drug Sensitivity in Cancer database, and their efficacy was determined. Chemotherapeutic drugs like dasatinib and IWP-2 were more suitable for the patients in the high-risk group (**Figure 7D–G**), whereas the other eight chemotherapeutic drugs were more suitable for the patients in the low-risk group (**Supplementary Figures 6A–H**). To provide more avenues for LLPSRS-based therapies, the Cancer Therapeutics Response Portal (CTRP) and Profiling Relative Inhibition Simultaneously in Mixtures (PRISM) were used to analyze LLPSRS’s correlation with chemotherapeutic agents. Five CTRP (e.g., dasatinib and fluvastatin) and five PRISM drugs (e.g., epothilone-b and tosedostat) were more suitable for the treatment of patients in the high-risk group (**Figure 7H**), whereas four CTRP (e.g., apicidin and brefeldin A) and nine PRISM drugs (e.g., poziotinib and RITA; **Supplementary Figure 6I**) were more suitable for the treatment of patients in the low-risk group. Further, the data obtained from the CellMiner (33) were analyzed, and 20 negative and 12 positive LLPSRS-related drugs were identified. Therefore, all patients with different LLPSRSs may respond to different chemotherapeutic drugs (**Figure 7I**, **Supplementary Figure 6J**).

### Utility and robustness of liquid–liquid phase separation-related risk score for predicting immunotherapeutic benefits

**Figure 8:**
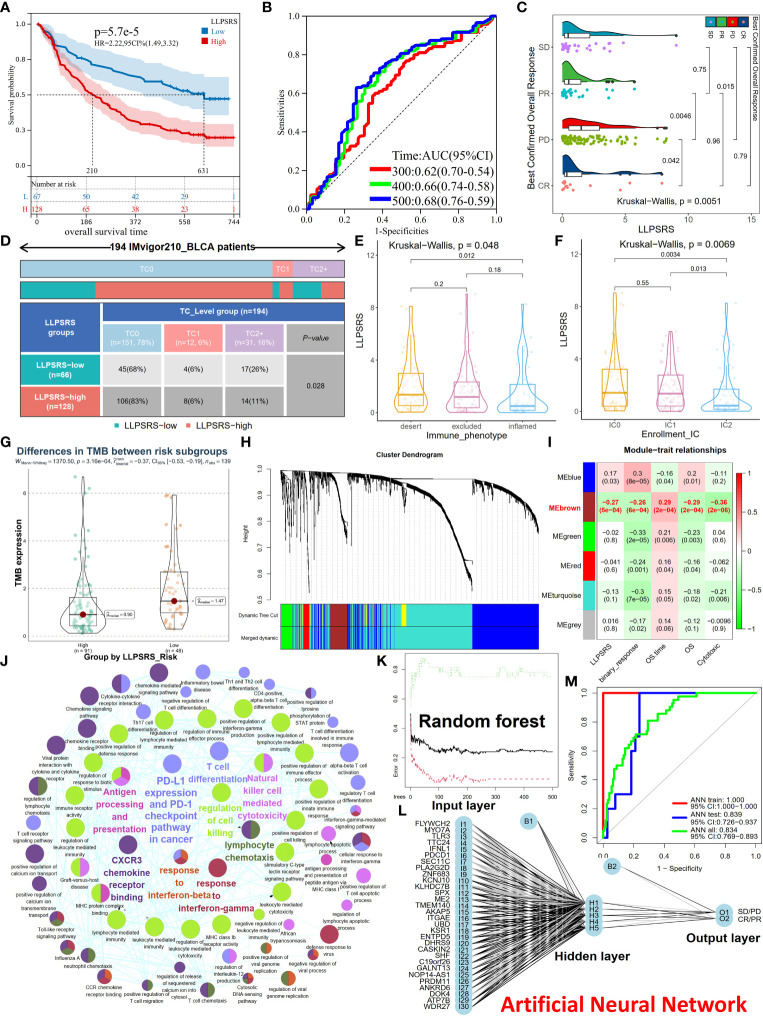
Utility and robustness of the LLPSRS for speculating immunotherapeutic benefits in the IMvigor210 cohort. **(A, B)** KM and ROC curves of LLPSRS in predicting OS. **(C)** Difference of LLPSRS among different responses to ICB. **(D)** Proportions of two subgroups in different TC levels (PD-L1 expression on tumor cells) (chi-square test). **(E, F)** Differences of LLPSRS among different IC levels (PD-L1 expression on immune cells) or immunophenotypes. **(G)** Difference of TMB between high- and low-risk subgroups. **(H)** The branches of cluster dendrogram correspond to the different gene modules. Each leaf on the cluster dendrogram corresponds to a gene, and the colored row represents a color-coded module that contains a group of highly connected genes. **(I)** Correlation coefficients of WGCNA gene modules with LLPSRS, cytotoxic, binary response, OS, and OS time. **(J)** Eigengenes’ annotations of GO terms and KEGG pathways. **(K)** The influence of number of decision trees on the error rate. The X-axis represents the number of decision trees, and the Y-axis indicates the error rate. **(L)** Our ANN can predict patients’ responses to ICB. **(M)** Model classification performances are displayed by ROC–AUC values in the IMvigor210 cohort, training, and validation sets. LLPSRS, liquid–liquid phase separation-related risk score; KM, Kaplan–Meier; ROC, receiver operating characteristic; OS, overall survival; ICB, immune checkpoint blockade; TMB, tumor mutational burden; WGCNA, Weighted Gene Co-expression Network Analysis; GO, Gene Ontology; KEGG, Kyoto Encyclopedia of Genes and Genomes; ANN, artificial neural network; AUC, area under the receiver operating characteristic curve.

Immunotherapy was regarded as an epoch-making breakthrough. LLPSRS’s correlation with the immunosuppressive milieu and TMB was observed, so LLPSRS could likely play a role in predicting patients’ response to ICB. We analyzed the association between LLPSRS and sensitivity to ICB in the IMvigor210 cohort. The patients in the low-risk subgroup had a longer life span and demonstrated prolonged survival as compared to those in the high-risk subgroup (log-rank test, p< 0.0001, **Figures 8A, B**). We also discovered that the progressive disease subgroup, in which TIICs exerted a faint effect, had the highest LLPSRS (Kruskal–Wallis test, p< 0.01, **Figure 8C**). Together, these results show a positive correlation between LLPSRS and irAEs, thus suggesting that patients with lower LLPSRS may respond better to ICB.

PD-L1 expression is an important biomarker for ICB responsiveness. Hence, we analyzed the correlation between TC (PD-L1 located on tumor cells), IC (PD-L1 located on immune cells), immunophenotype (desert, inflamed, and excluded), and LLPSRS. In the patients in the low-risk subgroup, TC2+ was the most abundant TC, thus indicating that the patients had higher sensitivity to ICB treatment (chi-squared test, p< 0.05, **Figure 8D**). Next, the patients in the IC2 subgroup or immune-inflamed type had the lowest LLPSRS (Kruskal–Wallis test, p< 0.01, p< 0.05, respectively, **Figures 8E, F**). A significant difference was observed in TMB between the high- and low-risk subgroups (Wilcoxon test, p< 0.001, **Figure 8G**). Further, the predictive ability of the combined TMB and LLPSRS was better as compared to LLPSRS and TMB alone (**Supplementary Figure 7A**). Therefore, for those patients treated with ICB, LLPSRS could effectively predict individual responses and immunosuppressive properties.

The outcomes of patients treated with ICB were better; however, not all patients experienced durable response to ICB treatment. Hence, there is an urgent need to identify eligible patients. Further, a diagnostic model was created based on LLPSRS and cytotoxicity using ANN. Weighted Gene Co-expression Network Analysis (WGCNA) was used to predict the patient’s response to ICB and determine its relevance with LLPSRS. WGCNA was used to extract eigengenes from 1,758 DEGs between the complete response/partial response (CR/PR) and stable disease/progressive disease (SD/PD) subgroups, the optimal fitting degree was R^2^ = 0.85, and soft-thresholding was β = 3 (**Supplementary Figures 7B–F**). After the modules were merged with a disparity coefficient< 0.45 and overall gene counts< 30, the DEGs were divided into six modules (**Figure 8H**). Given that the correlation between clinical characteristics and module eigengenes (MEs) exists, a correlation was observed between MEbrown, (the main module) and LLPSRS, binary response, OS time, OS, and cytotoxicity (**Figure 8I**, Spearman’s rank test, p< 0.001). Eventually, in the MEbrown module, gene significance’s associations with module membership were analyzed, and the results of LLPSRS (rho = 0.79, p< 0.0001) and cytotoxicity (rho = 0.86, p< 0.0001) were significant (**Supplementary Figures 7G,H**); thus, 122 genes were regarded as eigengenes. The eigengenes enriched immune responses such as cytotoxicity, immunogenicity, PD-L1/PD-1 checkpoints, chemotaxis, T-cell differentiation, and interferon response, thereby confirming the successful extraction of eigengenes (**Figure 8J**).

To build a classifier for identifying ICB-sensitive patients, a combined prediction model was constructed using random forest (RF) and ANN algorithms based on eigengenes. Since the number of patients with BLCA treated with ICB was few, all samples from the IMvigor210 cohort were randomly split. In the training cohort, eigengenes were incorporated into the RF classifier, and 180 trees were selected as the parameter (**Figure 8K**). Next, the variable importance was measured, and the top 30 genes were selected (**Supplementary Figure 7I**). Based on these genes, an ANN model was created using the training cohort, which consisted of three layers: input (expression of 30 genes), hidden (scores and weights of genes), and output layer (SD/PD or CR/PR results) (**Figure 8L**). Finally, the ROC–AUC values for the ANN model in training, validation, and IMvigor210 cohorts were 1.000, 0.839, and 0.834, respectively. The accuracy of the model in predicting the response to ICB treatment in the training, validation, and IMvigor210 cohorts was 1.000, 0.750, and 0.720, respectively. Together, these results show the robustness and utility in predicting response to ICB treatment in patients with BLCA (**Figure 8M**).

## Discussion

LLPS plays an indispensable role in regulating the hallmarks of cancer. However, the different functions of LLPS in cancer are still unclear. Studies have shown that LLPS could aid in deciphering the heterogeneity of TME (34), exploring genomic alterations and transcriptional aberrations (35), and the impact of drug distribution into condensate (36). LLPS plays multiple roles in carcinogenesis; therefore, identifying LLPS-related biomarkers could offer important insights into defining tumor subtypes and evaluating the prognoses of patients. However, few studies have used LLPS in predicting the clinical outcomes of patients with BLCA. In this study, we have exclusively focused on BLCA and explored LLPS-related patterns to enhance our understanding of the role of LLPS in BLCA pathogenesis. We identified three LLPS-related subtypes of BLCA and distinct features, including cancer hallmarks and clinicopathological phenotypes. Based on individual heterogeneity, we calculated LLPSRS for integrative assessments. Further, we determined the correlation between LLPSRS and patient prognosis, genomic variations, epigenetic alterations, TME characteristics, and pharmacogenomics. Our results showed an inverse correlation between LLPSRS and the efficacy of ICB. We also constructed an LLPSRS-related eigengenes-based classifier using the RF and ANN algorithms to predict the patient’s sensitivity to ICB treatment.

Various studies have shown the involvement of LLPS in carcinogenesis and metastasis (37). Estrogen triggers MYC to form condensates in an LLPS-mediated manner, which increases VEGF expression and promotes angiogenesis (38). Purinosomes (39) and glucosomes (40) are liquid-like condensates, whereas the LLPS of glycogen can induce tumorigenesis (41). Moreover, the LLPS of transcriptional coactivators like YAP/TAZ plays a role in EMT and cancer aggressiveness (42, 43). In this study, we identified three LLPS clusters using NMF. The prognoses of patients in C2 were favorable. These patients had immunosuppressive TME, and the expression of oncogenes was low. The discrepancies in these results could be due to the small size of the tumor and the limitations of algorithms, which emphasize absolute quantity rather than relative quality. Compared to patients in C2, the prognoses of patients in C1 and C3 were poor, and they had dysregulated immune responses. Studies have demonstrated that the levels of TIICs play a vital role in mediating immune responses; however, the dense stroma prevents the entry of TIICs in tumors. As expected, the pathways enriched by C3 were associated with EMT and stromal activation, suggesting that patients had excluded immunophenotype and activated invasion–metastasis pathway. Meanwhile, the patients in C1 had basal subtypes and enriched the mismatch repair, MYC, and PI3K signaling pathways, which indicates an increase in cell proliferation and expression of oncogenes. Together, our results suggest crosstalk between LLPS and genes’ expression associated with TME features, which mediated BLCA’s prognosis and progression.

Given the subtypes’ multifaceted heterogeneities, we translated these qualitative clusters into quantitative LLPSRS to conduct integrative assessments of individual LLPS patterns in patients with BLCA. Of the 29 RSGs, EGF and SUPT6H maintain the functional integrity of biomolecular condensates as regulators. HNRNPH3 is a scaffold and plays an important role during condensate formation. LLPS of EGF alters SMAD3 phosphorylation to enhance EMT and stemness of cells (44), whereas SUPT6H influences the assembly of SGs and P-bodies (45). In addition, HNRNPH3 represents LLPS driving forces (46). Other RSGs are clients that bind to scaffolds and condensates, including P-bodies, SGs, nucleolus, and postsynaptic density.

Studies showed that genetic mutations or epigenetic alterations play a role in the occurrence and development of cancer pathogenesis and cachexia. The influence of LLPS on genetic mutations or epigenetic alterations would aid in enhancing our understanding of carcinogenesis. For example, ubiquitin-tagged p62 cannot be degraded due to the mutant’s LLPS, which leads to Paget disease (47). SPOP mutants inhibit LLPS of substrates (48), and SHP2 mutants recruit wild-type SHP2 to condensates, which triggers carcinogenesis (49). YTHDC1 is an m^6^A reader and undergoes LLPS, which destabilizes mRNA and promotes tumorigenesis (50). IDRs in chromatins and enzymes undergo LLPS, leading to chromatin compartmentalization (51). In this study, the patients with high LLPSRS had distinct mutation patterns. Interestingly, an inverse correlation was observed between LLPSRS and TMB. However, a correlation between LLPS epigenetic aberrations was observed, thereby implying hyperprogression on treatment with ICB. Higher LLPSRS was associated with DNA hypermethylation and various RNA-editing regulators, which indicates that LLPS plays an important role in epigenetic regulation. Furthermore, AS of several DEGs led to refractory cachexia between the two subgroups. Our results show a correlation between epigenetic alterations and LLPSRS, which results in discrepancies in clinical outcomes.

Due to the target-independent physicochemical features, the chemotherapeutic drugs can be selectively distributed among distinct condensates, which reduces the efficacy of the drugs (52). For undruggable proteins, mediating their condensates offered intriguing avenues for antineoplastics. LLPS in LINP1 inhibits DNA repair and induces chemoresistance (53). An inhibition in MED1 expression could enhance the accumulation of its condensates on MYC genes, which increases the efficacy of tamoxifen (52). Furthermore, SHP2 allosteric inhibitors can disrupt condensates of SHP2 mutants (49), and NCOA1 LLPS can be attenuated by Elvitegravir (54). Further, the correlation between LLPSRS and the signatures of adjuvant therapies was investigated, which could aid in designing personalized treatment for patients with BLCA. The patients with high LLPSRS were sensitive to EGFR-targeted therapies, radiotherapy, and neoadjuvant chemotherapy. Patients with low LLPSRS may benefit from therapies targeting oncogenic pathways (like the FGFR3 and WNT signaling pathways) and ICB therapy. Further, LLPSRS was used to determine the efficacy of anticancer drugs by predicting chemosensitivity. Together, these results indicate the potential role of LLPSRS in predicting personalized treatment at the pharmacogenomics level; however, additional studies are required to study the underlying mechanism.

Various studies have divided TME into three immunophenotypes such as inflamed, excluded, and desert; however, spatiotemporal regulation of TME immunophenotypes is still unclear. A study has shown that YAP’s LLPS causes ICB hyperprogression induced by IFN-γ (55); therefore, the further correlation between LLPSRS and TME should be explored. A study has shown that LLPS of cGAS can activate innate immune responses and cGAMP production (56). However, STING forms spherical condensate to inhibit cGAMP signaling, which triggers innate immune responses (57). This indicates that LLPS plays a dual role in the cGAS-STING pathway. LLPS of NLRP6 promotes the secretion of IL-1β and IL-18 and induces pyroptosis (58). LLPS is involved in both innate and adaptive immune responses. T-cell receptor stimulators undergo LLPS; however, CD45 is excluded from condensates to ensure T-cell activation (59). In this study, the patients with lower LLPSRS had strong immune responses; hence, they were more likely to benefit from ICB treatment. By the IMvigor210 cohort, we directly attested that they responded better to ICB, and LLPSRS was a robust metric for evaluating individual responses. Only a few patients respond to ICB treatment, which prolonged their survival; therefore, LLPSRS could be used to identify patients with BLCA who could benefit from ICB. A total of 122 LLPSRS-relevant eigengenes were screened using WGCNA, and a prediction n classifier was constructed using the RF and ANN algorithms. The classifier was robust and could effectively predict the response of patients to ICB therapy.

However, our study has a few limitations. Firstly, we analyzed cross-sectional and retrospective data; hence, additional studies with prospective multi-center cohorts are required to validate our findings. Next, we explored the role of LLPS-related genes at the macroscopic level; however, these genes are involved in functions that are independent of LLPS. Hence, these LLPS-related genes are insufficient to determine the crosstalk between LLPS patterns and other characteristics. In this study, we explored the heterogeneity in TME and quantified LLPS patterns. However, we did not explore the intratumor heterogeneity in a single patient. We divided the patients into clusters based on the median LLPSRS as the cutoff value; however, we performed correlation analyses to reduce the bias. Since the number of patients with BLCA treated with ICB was few, we carried out no extra external validations. However, we carried out internal validations to compensate for this shortcoming. Furthermore, we used qRT-PCR to evaluate LLPSRS in clinical practice; hence, follow-up studies are required for designing genetic testing kits. Among RSGs, HNRNPH3 and NSUN5 play an important role in BLCA based on *in vitro* results; hence, we further analyzed them. Nevertheless, our results shed light on different subtypes of BLCA, which could aid in designing personalized treatment and provide insights into guidelines for clinical application.

## Conclusions

Our results revealed the underlying heterogeneity of tumors and the impact of LLPS on the biological functions of BLCA at the multi-omics level. We categorized the patients with BLCA into three subtypes. These patients had different prognoses, TME characteristics, cancer hallmarks, etc. We also calculated the LLPSRS using various algorithms, which could identify intricate LLPS patterns and develop their robustness from multifaceted dimensions. Encouragingly, in the era where ICB sheds new light on anticancer treatment, our binary classifier could effectively predict patients’ response to ICB, which would aid in designing personalized therapeutic strategies for patients. Our results aid in uncovering the complexities of LLPS. We developed algorithms to categorize patients with BLCA based on LLPS patterns, which will aid in developing personalized therapeutic strategies and shed light on personalized precision medicine.

## Data availability statement

All databases generated or analyzed for this study are included or have their accession numbers included in the article.

## Author contributions

SL designed this research. LS and SL organized the processing flow. LS, XY, SW, XT, GL, X-PL, HH, DW and CC completed the whole analytic process of this study. LS and XY organized and presented the results. LS contributed to the writing of the manuscript. All authors contributed to the article and approved the submitted version.

## Funding

This work was supported by the Zhongnan Hospital of Wuhan University Science, Technology and Innovation Seed Fund (znpy2019077) and Translational Medicine and Interdisciplinary Research Joint Fund of Zhongnan Hospital of Wuhan University (Grant No. ZNJC202232).

## Acknowledgments

We are grateful for TCGA and GEO databases developed by the National Institutes of Health (NIH), the cBioPortal website developed by the Memorial Sloan Kettering Cancer Center (MSK), and the ArrayExpress database developed by EMBL’s European Bioinformatics Institute.

## Conflict of interest

The authors declare that the research was conducted in the absence of any commercial or financial relationships that could be construed as a potential conflict of interest.

## Publisher’s note

All claims expressed in this article are solely those of the authors and do not necessarily represent those of their affiliated organizations, or those of the publisher, the editors and the reviewers. Any product that may be evaluated in this article, or claim that may be made by its manufacturer, is not guaranteed or endorsed by the publisher.

## Supplementary material

Supplementary Figure 1
**(A, B)** Consensus map of NMF based on 586 prognostic LLPS-related genes when k = 2/3/4/5; and NMF rank survey helped us to choose the appropriate rank value. **(C–E)** Consensus map of NMF when k = 2/3/4/5 in Meta-BLCA samples; and NMF rank survey helped us to choose the appropriate rank value. **(F)** KM curve exhibited significantly different OS among 3 LLPS clusters in Meta-BLCA cohort. (log-rank test, P< 0.001, samples that OS time > 4000 days were deleted). **(G–I)** Differences in 12 BLCA, 20 SA-MR-IR, and 19 oncogene signatures among LLPS clusters. The line in the box represented the median value and the asterisk represented the P value (“*” P< 0.05; “**” P< 0.01; “***” P< 0.001; “****” P< 0.0001), while the statistical analyses were performed by the Kruskal-Wallis test.

Supplementary Figure 2
**(A)** Venn diagrams showed overlaps of 470 DEGs (|log2FC| > 1, P< 0.01) among the three LLPS clusters. **(B, C)** Consensus map of NMF based on 197 prognostic LLPS-related DEGs when k = 2/3/4/5; and NMF rank survey helped us to choose the appropriate rank value. **(D–F)** Differences in 12 BLCA, 20 SA-MR-IR, and 19 oncogene signatures among three DEG clusters. The line in the box represented the median value and the asterisk represented the P value (“*” P< 0.05; “**” P< 0.01; “***” P< 0.001; “****” P< 0.0001), while the statistical analyses were performed by the Kruskal-Wallis test.

Supplementary Figure 3
**(A, B)** The relation between lambda values and variable coefficients or partial likelihood deviance in the LASSO regression. **(C–E)** Patients were divided into high- and low-risk subgroup in the training set, TCGA-BLCA cohort, and validation set; survival status of patients in two subgroups; heatmap of 29 RSGs. **(F–I)** KM curves for LLPSRS in the external validation sets (Meta-BLCA, GSE13507, GSE31684, E-MTAB-4321 cohorts). **(I–N)** Comparison of LLPSRS with other six prognostic signatures in terms of DCA, C-index and ROC-AUC values at 1, 3, 5 and 7 years.

Supplementary Figure 4
**(A)** A histogram of differential transcript levels of NSUN5, DAD1, XPO5, HMG20B and DHX16 in BLCA and normal urothelial cell lines. **(B, C)** Univariate Cox analysis and Net reduction analysis of LLPSRS and 7 clinical traits for the prognostic prediction in the TCGA-BLCA cohort. **(D–G)** The ROC curves for LLPSRS and seven clinical traits at 1, 3, 5 and 7 years. **(H)** The CNV variation frequency of 29 RSGs in the TCGA-BLCA cohort. The height of the column represented the alteration frequency, and the deletion was referred to as green dot while the amplification was referred to as red dot. **(I, J)** LLPSRS were inversely correlated with TMB and purity in the TCGA-BLCA cohort. **(K, L)** The multi-omics mutation and interconnection landscape of 29 RSGs.

Supplementary Figure 5
**(A)** KEGG annotation of 240 DNA methylation-relevant driver genes. The different colors represented the different terms or pathways. **(B)** Heatmap showed the DNA methylation levels of top 35 hypermethylated or hypomethylated genes. **(C–E)** Differential expression of RNA modification regulators between high- and low- risk subgroups. Color code of the regulators indicated corresponding modification type. **(F)** Network diagram showed that 786 of DEGs were discovered to have co-expression relations with 71 RNA editing regulators with |Pearson’s r| > 0.3 and p< 0.0001 as the threshold. **(G)** KEGG annotation of 786 RNA editing-relevant DEGs. The different colors represented the different terms or pathways. **(H)** KEGG annotation of 161 DEGs with AS. The different colors represented the different terms or pathways. **(I)** The splicing regulatory network between the prognostic AS events of DEGs and relevant splicing factors.

Supplementary Figure 6
**(A–H)** Paclitaxel, Gemcitabine, AZD4547, 5-Fluorouracil, Dactinomycin, Epirubicin, Oxaliplatin, Lapatinib showed lower IC50 value in low-risk subgroup *via* GDSC. **(I)** 4 CTRP-related and 9 PRISM-related compounds were identified by correlation between LLPSRS and AUC value. **(J)** 20 Cellminer-related potential drugs for patients with lower LLPSRS were identified by correlation between LLPSRS and G150 value. The line in the box represented the median value and the asterisk represented the P value (“*” P< 0.05; “**” P< 0.01; “***” P< 0.001; “****” P< 0.0001), while the statistical analyses were performed by the Mann-Whitney and Spearman correlation test.

Supplementary Figure 7
**(A)** ROC curve of LLPSRS in patients treated with anti-PD-L1 immunotherapy. The combination of TMB and LLPSRS improved predictive power compared with that of TMB or LLPSRS alone. **(B)** Euclidean distance between samples were calculated by the “dist” function. **(C)** Relation between the scale independence (R^2^) and mean connectivity with the soft threshold (β). **(D)** Eigengene adjacency heatmap. **(E)** Network heatmap plot. **(F, G)** Scatter plot of module eigengenes in the brown module. **(H)** Results of the Gini coefficient method in RF classifier. The X-axis indicated the genetic variable, and the Y-axis represented the importance index.
