# Supplementary material for: Identification of molecular subtypes based on liquid–liquid phase separation and cross-talk with immunological phenotype in bladder cancer
Source: Front Immunol. 2022 Nov 28;13:1059568. doi: 10.3389/fimmu.2022.1059568 (PMC9742536; doi:10.3389/fimmu.2022.1059568)
Supplement: Supplementary file 1 [file DataSheet_1.docx]

**Materials and Methods**

**Data source and processing**

Publicly available (public databases or supplementary data from the published researches) gene expression datasets for BLCA were searched and collected. BLCA gene expression datasets with integral phenotype annotation were downloaded, while the samples without prognosis information were excluded from further investigation. Original data of LLPS-related genes were extracted from the integrative database DrLLPS (<http://llps.biocuckoo.cn/>) that incorporated 9,285 LLPS-associated proteins (150 scaffolds, 987 regulators and 8,148 potential clients), all of which were verified by previous experiments (1). On account of the fact that the research object was Homo sapiens, so 5652 LLPS-relevant proteins experimentally identified in other eukaryotic species were eliminated, and a total of 3633 proteins were downloaded. A total of six comprehensive cohorts (TCGA-BLCA, GSE13507, GSE31684, GSE188715, E-MTAB-4321 and IMvigor210) were utilized in our study. We enrolled the patients with complete annotation of prognosis, and those with an overall survival time of < 90 days were excluded due to other possible causes of loss to follow-up. The Gene Expression Omnibus (GEO) (<https://www.ncbi.nlm.nih.gov/geo/>) were used to obtain the original data from GSE13507, GSE31684 and GSE188715 datasets (2). Next, transcriptome information that deposited in the TCGA-BLCA cohort (FPKM and Counts value) (3), accompanied by corresponding phenotype, DNA methylation, SNP and CNV data, were extracted from the UCSC Xena Browser (https://xena.ucsc.edu/) (4). Then, the raw data of E-MTAB-4321 cohort was collected from the ArrayExpress database in EMBL-EBI (https://www.ebi.ac.uk/arrayexpress/) (5). Eventually, IMvigor210 cohort, which was designed to investigate response to PD-L1 blockade with atezolizumab in metastatic urothelial cancer (mUC), was also retrieved in our study using R package “IMvigor210CoreBiologies” (6). Furthermore, all expression data was normalized such as log2(FPKM+1) for subsequent multi-database analysis. For GSE13507, GSE31684 and GSE188715 cohorts, “normalizeBetweenArrays” function in R package “limma” were performed to carry out the background adjustment and quantitative normalization (7), and the Rank-in (<http://www.badd-cao.net/rank-in/>) algorithm enabled integrative analysis across microarray and RNA-seq was further used to remove the batch effect of BLCA-meta cohort (8).

**Identification of LLPS Subtypes**

Initially, 3633 LLPS-associated proteins were selected and 586 LLPS-related genes that associated with prognosis were retained via univariate Cox analysis in the BLCA-TCGA cohort (P < 0.01). On the basis of these 586 genes’ expression level, we utilized nonnegative matrix factorization (NMF) clustering analysis to identify the molecular subtypes with the R package “NMF”. Specifically, NMF was an effective technique for reducing dimensionality of complex data sets. By factorizing the original matrix into two non-negative matrices, NMF was competent to identify potential characteristics in gene expression profiles. Deposition of the original matrix was repeatedly performed, and its output was aggregated to obtain uniformly clustered PTC samples. The optimal number of subtypes was selected in accordance with cophenetic, dispersion and silhouette coefficients. Three LLPS subtypes, were obtained and designated as LLPS cluster C1/C2/C3 respectively. To further identify the robustness and reliability of LLPS subtype, principal component analysis (PCA) was conducted, and the classification result was reproducible in BLCA-meta cohort.

**Term, Pathway, Signature and Functional Enrichment Analysis**

In order to ascertain biological mechanisms’ heterogeneity among different LLPS subtypes, and gene set variation analysis (GSVA), identified as an nonparametric unsupervised method (9), was executed based on hallmark gene set that was downloaded from the MSigDB database in the form of “h.all.v7.5.1.symbols” (10). Single-sample gene set enrichment analysis (ssGSEA) were performed using the R package “GSVA” (9), and our study dabbled in multitudinous multiple gene signatures acquired from Supplementary Material or online platforms of several published studies (11-14). The enrichment score of 34 TME infiltration cells was estimated using the ssGSEA algorithm; and the status of anti-cancer immunity that conceptualized a seven-step cancer-immunity cycle, was quantified in “Tracking Tumor Immunophenotype” (TIP) online platform (14). Furthermore, we conducted over-representation analysis of the Gene Ontology (GO) terms and Kyoto Encyclopedia of Genes and Genomes (KEGG) pathways by using the R package "clusterProfiler 4.0" (15), and visualization of corresponding results was completed by the R package “GOplot” (16). Additionally, we also performed the over-representation analysis of GO terms and KEGG pathways using the Cytoscape (17) plugin “ClueGO” (18), the different colors represented the different terms or pathways. To explore the underlying hallmark pathways between the high- and low-risk subgroups, we ran gene set enrichment analysis (GSEA) by means of the hallmark gene sets (19).

**Comprehensive analysis of DEGs from LLPS subtypes in BLCA**

In order to further unravel the underlying heterogeneity of the LLPS subtypes in BLCA, we performed differential analyses via the “DESeq2” package of R software (20). 470 differentially expressed genes (DEGs) among three LLPS subtypes (|log2foldchange| > 1, P < 0.01) were identified, and they were annotated by the R package “clusterProfiler” to conduct over-representation analysis of GO terms and KEGG pathways (15). Moreover, the visualization of DEGs’ enrichment analysis was completed by the R package “GOplot” (16). To further identify the probable classification of LLPS subtype and uncover more mechanisms, 197 prognostic DEGs were identified as candidate genes and they would be used in the subsequent analysis of DEGs’ clustering by conducting univariate Cox regression analysis (P < 0.01). NMF was performed based on the expression of 197 prognostic DEGs (P < 0.01) in TCGA-BLCA cohort. After full-scale and well-thought-out consideration, k = 3 was chosen as the optimal k value of cluster number depending on the cophenetic correlation coefficients, and it can be seen that the three subtypes had obvious boundaries when k = 3, suggesting a stable and appropriate clustering for the TCGA-BLCA cohort.

**Derivation and Construction of LLPSRS**

In the TCGA-BLCA cohort, all samples were randomly split into the train (n = 270) and test (n = 112) sets by a ratio of 7:3, afterwards 424 prognostic genes were discovered out of the aforementioned 586 LLPS-related genes using multivariate cox regression analysis (P < 0.05) in the train set. Next, by employing the least absolute shrinkage and selection operator (LASSO) Cox regression algorithm (21) to minimize the risk of over-fitting using the R package “glmnet” (22), 60 powerful candidate genes with optimal penalty coefficient were determined. Then, by performing multivariate Cox analysis with a Cox hazards regression model through a stepwise variable-selecting procedure, 29 hub genes, along with their correlative coefficients, were further highlighted from the abovementioned 60 genes. Finally, a LLPS-related risk score (LLPSRS) was established through multiplying the non-zero coefficients with the corresponding gene expression for predicting BLCA patients’ prognostic outcomes. In the train set, 270 patients were stratified into the high-risk (n = 135) or low-risk (n = 135) subgroups according to their LLPSRS and calculated LLPSRS’s median cut-off, and patients in the test set were also assigned into the high-risk (n = 57) or low-risk (n = 55) subgroups based on the train set’s cut-off value. The distribution changes of survival differences among the different LLPS subtypes, DEG subtypes and risk groups were visualized with an alluvial diagram. Then we further explored the relationship between the LLPSRS and LLPS or DEG subtypes, and the Kruskal-Wallis test revealed a significant difference on LLPSRS among LLPS or DEG subtypes.

**Validation and Versatility of LLPSRS**

In the train, test set and TCGA-BLCA cohort, we analyzed the relationship between different LLPSRS and patients' follow-up time, events and changes in the expression of each gene, and it was observed that the OS rate of patients decreased significantly with the increase of LLPSRS. In addition, we further evaluated its performance in external BLCA cohorts (the GSE13507, GSE31684, GSE188715, LUAD-meta cohort), and patients were classified into high- or low-risk subgroups using the optimal cut-off value of LLPSRS obtained by the R package ‘Survminer’. In order to determine the area of under curve value (AUC) and assess the predictive power of the LLPSRS, survival analysis was carried out for two subgroups in the form of Kaplan-Meier curves, and the receiver operating characteristic (ROC) curve analysis was performed by the R package “pROC”. Furthermore, in the E-MTAB-4321 cohort, samples were classified into the high- or low-risk subgroups according to LLPSRS’s median value, and the PFS rate of the high-risk subgroup was significantly lower than that of the low-risk subgroup. Finally, we retrieved six prognostic algorithms published within the last year, and we compared their prognosis prediction ability with LLPSRS through the decision curve analysis (DCA), concordance index (C-index), and ROC curve analysis (11, 23-27). Since LLPSRS was associated with high malignancy, we sought to investigate whether LLPSRS remained a clinically independent prognostic factor and performed better than traditional clinical features for predicting prognosis in BLCA through univariate and multivariate Cox regression analyses. Combined with other clinical characteristics, including age, gender, grade, AJCC T, AJCC N, AJCC M and pathological tumor stage, were enrolled as covariates to carry out the analysis. Moreover, in the TCGA-BLCA cohort, the DCA, net reduction, and ROC curves analysis were employed to compare LLPSRS with other clinical parameters in terms of prognostic predictive capability. Further univariate cox analysis was respectively performed on subgroups with different characteristics to compensate for bias caused by different clinicopathological trait stratifications. According to the inference that bladder cancer’s molecular subtypes could promote personalized anticancer treatment for patients (28), and several conventional molecular subtype classification systems (29), including the UNC (30), TCGA (31), MDA (32), Lund (33), CIT (34), Consensus (35), and Baylor (36) subtypes, were correlated with LLPSRS, we provided an overview of the differences in clinicopathological landscape between high- and low-risk subgroups in the TCGA-BLCA cohort.

**Genomic Alterations and Epigenetic Variations Analysis**

To investigate the somatic mutations regarding LLPSRS, the “MuTect2 Variant Aggregation and Masking” data of TCGA-BLCA cohort was retrieved, and the waterfall plots manifesting the mutation landscape were depicted by the R package “maftools” (37). To obtain TMB values reflecting total mutation numbers, we calculated the total number of non-synonymous mutations using 38MB as estimate of exome size, and it was divided by the size of target genes coding region. Besides, somatic copy number variations (CNV) calling was performed with GISTIC2.0 algorithm (38), and the R package “Rcircos” was used to visualize the positions of CNVs on the chromosome (39). Moreover, as potential indices for the ICB response and formation of neoantigenic epitopes, tumor mutation burden (TMB) and purity data for each sample were obtained (40), and their correlation with LLPSRS were explored by Spearman’s coefficient correlation test. In addition, the interconnection and mutation landscape of the 29 genes that constituted LLPSRS were visualized using cBioPortal (41). The DNA methylation profiles “Illumina Human Methylation 450” was obtained, and the differentially expressed CpG islands (DECGs) and methylated genes (DMGs) between the high- and low-risk subgroups were identified by Wilcox non-parametric test (P < 0.05), while 1416 DEGs (|log2foldchange| > 1, P < 0.01) were identified via the “DESeq2” package of R software. Next, correlation analysis between DEGs and corresponding DMGs was performed using the R package “MethylMix 2.0” (42), and 240 DEGs whose expression could be influenced by corresponding DNA methylation levels were identified as driver genes (P < 0.0001, |Pearson’s r| > 0.4). 26583 DECGs were used to depict the DNA methylation landscape in a heatmap, and the top 35 hypermethylated or hypomethylated genes’ DNA methylation levels were visualized. Since RNA editing and alternative splicing acted as key regulators in cancers’ development, we explored the association between LLPSRS and post-transcriptional regulation. We curated 95 RNA editing regulators, 10 for N1-methyladenosine (m^1^A), 13 for 5-methylcytidine (m^5^C), 16 for N6-methyladenosine (m^6^A), 28 for N7-methylguanosine (m^7^G), 22 for Alternative polyadenylation (APA), and 6 for Adenosine to inosine (A-to-I). Moreover, we downloaded the TCGA-BLCA cohort’s alternative splicing data from TCGASpliceSeq (43). Afterwards, 61 prognosis-related AS events were selected (univariate Cox regression, P < 0.05), and 37 of them were discovered to have co-expression relationships with 22 splicing factors with |Pearson’s r| > 0.3 and p < 0.0001 as the threshold, finally the interaction networks between the prognostic AS events and splicing factors were constructed.

**Evaluation of TIME Cells Infiltration and Immunological Characters**

In order to evaluate the abundance of TIME-infiltrating cells and their relationship with LLPSRS, we introduced and adopted ten independent algorithms of appraisal, including TIMER (44), QuanTIseq (45), TIP (14), xCell (46), CIBERSORT-ABS, CIBERSORT (47), MCP-counter (48), ImmuCellAI (49), TISIDB (50) and EPIC (51), based on the RNA-seq data in TCGA-BLCA cohort. Additionally, from literatures we curated a total of 23 immune checkpoints, 24 components of HLA class, 16 immunomodulators, and 17 chemokines and chemokine receptors in order to further explore molecular immunological characteristics of LLPSRS.

**Sensitivity of** **Chemotherapeutic Treatment**

Integrated cell line datasets with corresponding drug sensitivity information were extracted and downloaded from the Genomics of Drug Sensitivity in Cancer (GDSC) (https://www.cancerrxgene.org/) database (52), and sensitivity of chemotherapeutic treatment of BLCA patients were inferred using oncoPredict (53). In terms of recommended drug selection, predicted the half-maximal inhibitory concentration (IC50) values for 12 kinds of drugs represented differential expression between two subgroups: 4 drugs might be appropriate agents to treatment of patients with higher LLPSRS, while other drugs were identified to be suitable for patients with lower LLPSRS (Wilcox non-parametric test, P < 0.05). The Cancer Therapeutics Response Portal (CTRP: <https://portals.broadinstitute.org/ctrp/>) and the profiling of relative inhibition simultaneously in mixtures (PRISM: https://depmap.org/repurposing/) (54, 55) database were harnessed to analysis the drug sensitivity association between LLPSRS and chemotherapeutic agents. We extended special thanks to Chen Yang for his support with R-script design and analysis methodologies (56), and Paul Geeleher for development of the R package “pRRophetic” (57). Through difference analysis in drugs’ efficacy between the high- and low-risk subgroups (upper and lower deciles), the compounds with lower area under the curve (AUC) values were selected, respectively (P < 0.05). Then, Spearman correlation analysis between drugs’ AUC value and LLPSRS was conducted to screen out compounds with negative correlation coefficients, respectively (P < 0.01, Spearman's r < -0.2). Moreover, the CellMiner (https://discover.nci.nih.gov/cellminer/), a user-friendly tool that organized and stored standardized data including multiple types of molecular features at pharmacogenomics levels, were used to identify the LLPSRS’s association with drugs’ sensitivity. Our calculating model was performed to screen and retain potential drugs by analyzing GI50 data (IC50 data revised by the NCI) of over 20,000 compounds and 59 cancer cell lines’ transcriptome data embodied in the CellMiner database. Next, only 792 drugs approved by FDA or under clinical trials were incorporated into our study so as to apply LLPSRS to actual practice as soon as clinically possible. Then, Spearman correlation analyses were performed on the GI50 data of 792 drugs and LLPSRS of abovementioned cell lines, and thus 93 drugs were significant (P < 0.05), including 32 drugs positively associated and 61 drugs negatively associated. Among them, 12 positively and 20 negatively associated drugs, suggesting that patients with lower or higher LLPSRS were more sensitive to these drugs respectively, were selected to visualize in the form of scatter plots.

**Utility and Robustness of LLPSRS in ICB Cohort**

We sought to illustrate the relationship between LLPSRS and ICB treatment response in patients with metastatic urothelial cancer (mUC), and the IMvigor210 cohort, which consisted of abundant patients treated with anti-PD-L1 immunotherapy (atezolizumab) and their matching complete clinical or transcriptome data, was downloaded and preprocessed by the R package “IMvigor210CoreBiologies” for our subsequent investigation (6, 58). After omitting patients who didn’t suffer from bladder cancer or had no information about overall survival, 195 mUC samples were finally screened out and included in our study. We further evaluated the LLPSRS performance in the IMvigor210 cohort, and 195 patients were classified into high- (n = 128) or low-risk (n = 67) subgroups using the optimal cut-off value of LLPSRS obtained by ‘Survminer’.

**Extracting ICB response and LLPSRS-relevant** **Eigengenes via WGCNA**

For better predicting patients’ response to Immunotherapy and understanding its association with LLPSRS and tumor-infiltrating immune cells (TIICs), we identified 1758 DEGs between group CR/PR and SD/PD in 168 patients with complete data of binary response to ICB (Wilcox test, P < 0.05). Then, Weighted Gene Co-expression Network Analysis (WGCNA) (59), which was commonly used to identify gene sets with highly synergistic changes, was performed on the expression data of 1758 DEGs in order to extract feature genes related to LLPSRS, TIICs, prognosis and ICB’s response. Since the “Cytotoxic” score analyzed by the ImmuCellAI platform well represented the TIL’s cytotoxic function, it was selected as the index of immune elimination ability. Next, to ensure the scale-free topology, the optimal fitting degree was selected as R^2^ = 0.85, and β = 3 was picked as soft-thresholding for subsequent adjacency calculation. After merging modules with the disparity coefficient < 0.45 and overall gene counts < 30, the1758 DEGs were split into six modules, and no-significant DEGs were clustered in the grey module. Then, by analyzing the associations between module eigengenes (ME) and clinical traits, the MEbrown was the main module related to LLPSRS, binary response, OS time, OS and Cytotoxic respectively (Spearman’s correlation test, P < 0.001). Eventually, scatterplot of the associations between Gene Significance (GS) and Module Membership (MM) in the brown module was visualized, and their correlation for LLPSRS (cor = 0.79, P = 2.9e-27) and Cytotoxic (cor = 0.86, P = 7.3e-37) were strikingly significant, so 122 genes in the brown module were regarded as key eigengenes. Immediately afterwards, enrichment analysis of GO terms and KEGG pathways were carried out to explore the underlying biological mechanisms of these eigengenes. Given the paucity of ICB treated cohort for bladder cancer with available transcriptome data, all 168 samples in IMvigor210 cohort were randomly split into the train (n = 120) and test (n = 48) sets by a ratio of seven to three.

**Establishment and Validation of the Novel Binary Classifier**

In order to build a novel binary classifier with the goal of distinguishing patients who might respond to ICB from patients who might present with severe irAEs, we constructed a joint prediction model of random forest and artificial neural network according to the 122 abovementioned ICB response and LLPSRS-related eigengenes. Given the paucity of ICB-treated cohorts for BLCA with available transcriptome data, all 168 samples in the IMvigor210 cohort were randomly split into the train (n = 120) and test (n = 48) sets by a ratio of 7:3. RF analysis was performed by the R package “randomForest”, and characteristic genes were screened out according to the point at which the error of cross validation was the least. In the train set, the eigengenes were input into the random forest classifier, and 180 trees, which revealed a stable error, were selected as the parameter based on the relationship plot between the cross-validation error, samples’ error value and the number of decision trees. Next, the variable importance of output results was measured by the score of Mean Decrease Gini, and top 30 genes with an importance greater than 0.5 were enrolled in subsequent analysis. Then, based on the expression of the top 30 genes, we used the train set to establish an ANN model by the R package “NeuralNetTools”. Artificial neural network consisted numerous processing units which thus formed a nonlinear and self-adjusting information processing system. In this classifier, the sum of the product of weight coefficients multiplied with corresponding expression levels of aforementioned genes was used as the response distinction score. Firstly, we preprocessed data by assigning scores to these genes: up-regulated genes above the median value were scored 1 while the rest were scored 0; similarly, down-regulated genes below the median value were scored 1 whereas the rest were scored 0. Next, the classifier model was constructed and it consisted of three dense layers: an input layer (contained 30 neurons, namely the expression of 30 genes), a hidden layer (with the scores and weights of genes) and an output layer (with the results for SD/PD and CR/PR samples). Then, the train set was used to determine the weights of candidate genes while the purpose of test or all set was to verify the classification efficiency of the model score constructed with gene expression and gene weight. Ultimately, we applied ROC curves to assure how precise the classification of ANN worked respectively on train, test and all set.

**RNA extraction and** **quantitative real‑time polymerase chain reaction (qRT‑PCR)**

The expression patterns of the HNRNPH3, CNOT11, NSUN5, DAD1, XPO5, HMG20B and DHX16 genes were investigated in matched cancerous and normal urothelial cell lines. The bladder cancer cell lines T24, 5637 and urothelial cell line SV-HUC-1 were obtained from the urological laboratory in Zhongnan Hospital of Wuhan University. According to the manufacturer instruction, T24 and 5637 cell lines were cultured in RPMI-1640 medium with 10% FBS, while SV-HUC-1 cell line were cultured in Ham's F-12K medium with 10% FBS and 1% Penicillin-Streptomycin Solution; and they were all incubated at 37℃ with 5% CO2. The HiPure Total RNA Mini Kit (Magen, China) was employed to extract total RNA from cell lines, and we harnessed NanoDrop to quantify the RNA, which was then reverse transcribed into cDNA by ReverTra Ace qPCR RT Kit (Toyobo, China). Eventually, we exploited qRT-PCR analysis of cDNA with iQTM SYBR® Green Supermix (Bio-Rad) in a final volume of 20 ml. The relative expression data was quantified via the 2-△△Ct algorithm and normalized to glyceraldehyde-3-phosphate dehydrogenase (GAPDH) as an internal control, and we evaluated the statistical significance by performing Student t-test and one-way ANOVA. HNRNPH3 primer: 5′-TGACAGAATGCGACGAGGAG-3′ (forward), 5′-TGGAAGAGGTATGGGAGGACA-3′ (reverse); CNOT11 primer: 5′-CCCTCCGCTCTCAGGATTTT-3′ (forward), 5′-CTTCAGTTAGCCTTGGCCGA-3′ (reverse); NSUN5 primer: 5′-CTCGTCTACTCCACGTGCTC-3′ (forward), 5′-CAAGCCTCACAGGCCAAAGC-3′ (reverse); DAD1 primer: 5′-AGTGTCTGTCATTTCGCGGT-3′ (forward), 5′-CCTAGCGGTTTGCCTGAGAA-3′ (reverse); XPO5 primer: 5′-CTGGATATGCTTGACGCGGA-3′ (forward), 5′-TGCTTCGTGTCTTTGTAAAGCCTC-3′ (reverse); HMG20B primer: 5′-GACCAAAACAAAGCGCGTGA-3′ (forward), 5′-CTCATCGTCCGCATCAAGGA-3′ (reverse); DHX16 primer: 5′-CCTGACAGGACAGGAGGAGA-3′ (forward), 5′-TGTGGCAACGAACATTGCTG-3′ (reverse); GAPDH primer: 5′-ACAACTTTGGTATCGTGGAAGG-3′ (forward), 5′-GCCATCACGCCACAGTTTC-3′ (reverse).

**Statistical Analysis**

The group variations can be well explored with Wilcoxon test, and for groups of more than two, Kruskal-Wallis test fitted well. The correlation analyses were performed by adopting appropriate Pearson's or Spearman’s correlation coefficient analysis as appropriate. Through Kaplan-Meier method, survival curves can be illustrated, then the log-rank test was used to analyze the difference in OS and DFS. Univariate and multivariate analyses were also used to elucidate the relationship between variables and the prognosis based on Cox proportional hazards regression (HR) model and 95% confidence intervals (CI). The accuracy of prognostic biomarkers and the net benefit on survival of BLCA patients between different signatures were respectively determined with ROC curves and decision curve analysis (DCA). Outcomes with P < 0.05 were defined to be statistically significant in comparisons between groups. Besides, we owed a debt of gratitude to Sangerbox for accommodating our demands of bioinformatics and statistical analysis and data visualization (60). The R 4.1.3 software (<https://www.r-project.org/>) was used for data processing and statistical analyses.

**REFERENCES**

1. Ning W, Guo Y, Lin S, Mei B, Wu Y, Jiang P, et al. DrLLPS: A data resource of liquid-liquid phase separation in eukaryotes. *Nucleic Acids Res* (2020) 48(D1):D288-95. doi: 10.1093/nar/gkz1027

2. Clough E, Barrett T. The gene expression omnibus database. *Methods Mol Biol* (2016) 1418(93-110. doi: 10.1007/978-1-4939-3578-9_5

3. Tomczak K, Czerwinska P, Wiznerowicz M. The Cancer Genome Atlas (TCGA): An immeasurable source of knowledge. *Contemp Oncol (Pozn)* (2015) 19(1A):A68-77. doi: 10.5114/wo.2014.47136

4. Goldman MJ, Craft B, Hastie M, Repecka K, McDade F, Kamath A, et al. Visualizing and interpreting cancer genomics data via the Xena platform. *Nat Biotechnol* (2020) 38(6):675-8. doi: 10.1038/s41587-020-0546-8

5. Brazma A, Parkinson H, Sarkans U, Shojatalab M, Vilo J, Abeygunawardena N, et al. ArrayExpress--a public repository for microarray gene expression data at the EBI. *Nucleic Acids Res* (2003) 31(1):68-71. doi: 10.1093/nar/gkg091

6. Mariathasan S, Turley SJ, Nickles D, Castiglioni A, Yuen K, Wang Y, et al. TGFbeta attenuates tumour response to PD-L1 blockade by contributing to exclusion of T cells. *Nature* (2018) 554(7693):544-8. doi: 10.1038/nature25501

7. Ritchie ME, Phipson B, Wu D, Hu Y, Law CW, Shi W, et al. Limma powers differential expression analyses for RNA-sequencing and microarray studies. *Nucleic Acids Res* (2015) 43(7):e47. doi: 10.1093/nar/gkv007

8. Tang K, Ji X, Zhou M, Deng Z, Huang Y, Zheng G, et al. Rank-in: Enabling integrative analysis across microarray and RNA-seq for cancer. *Nucleic Acids Res* (2021) 49(17):e99. doi: 10.1093/nar/gkab554

9. Hanzelmann S, Castelo R, Guinney J. GSVA: Gene set variation analysis for microarray and RNA-seq data. *Bmc Bioinformatics* (2013) 14(7. doi: 10.1186/1471-2105-14-7

10. Liberzon A, Birger C, Thorvaldsdottir H, Ghandi M, Mesirov JP, Tamayo P. The Molecular Signatures Database (MSigDB) hallmark gene set collection. *Cell Syst* (2015) 1(6):417-25. doi: 10.1016/j.cels.2015.12.004

11. Chen X, Chen H, Yao H, Zhao K, Zhang Y, He D, et al. Turning up the heat on non-immunoreactive tumors: Pyroptosis influences the tumor immune microenvironment in bladder cancer. *Oncogene* (2021) 40(45):6381-93. doi: 10.1038/s41388-021-02024-9

12. Hu J, Yu A, Othmane B, Qiu D, Li H, Li C, et al. Siglec15 shapes a non-inflamed tumor microenvironment and predicts the molecular subtype in bladder cancer. *Theranostics* (2021) 11(7):3089-108. doi: 10.7150/thno.53649

13. Luo Q, Vogeli TA. A Methylation-Based reclassification of bladder cancer based on immune cell genes. *Cancers (Basel)* (2020) 12(10). doi: 10.3390/cancers12103054

14. Xu L, Deng C, Pang B, Zhang X, Liu W, Liao G, et al. TIP: A web server for resolving tumor immunophenotype profiling. *Cancer Res* (2018) 78(23):6575-80. doi: 10.1158/0008-5472.CAN-18-0689

15. Wu T, Hu E, Xu S, Chen M, Guo P, Dai Z, et al. ClusterProfiler 4.0: A universal enrichment tool for interpreting omics data. *Innovation (Camb)* (2021) 2(3):100141. doi: 10.1016/j.xinn.2021.100141

16. Walter W, Sanchez-Cabo F, Ricote M. GOplot: An R package for visually combining expression data with functional analysis. *Bioinformatics* (2015) 31(17):2912-4. doi: 10.1093/bioinformatics/btv300

17. Shannon P, Markiel A, Ozier O, Baliga NS, Wang JT, Ramage D, et al. Cytoscape: A software environment for integrated models of biomolecular interaction networks. *Genome Res* (2003) 13(11):2498-504. doi: 10.1101/gr.1239303

18. Bindea G, Mlecnik B, Hackl H, Charoentong P, Tosolini M, Kirilovsky A, et al. ClueGO: A Cytoscape plug-in to decipher functionally grouped gene ontology and pathway annotation networks. *Bioinformatics* (2009) 25(8):1091-3. doi: 10.1093/bioinformatics/btp101

19. Subramanian A, Tamayo P, Mootha VK, Mukherjee S, Ebert BL, Gillette MA, et al. Gene set enrichment analysis: A knowledge-based approach for interpreting genome-wide expression profiles. *Proc Natl Acad Sci U S A* (2005) 102(43):15545-50. doi: 10.1073/pnas.0506580102

20. Love MI, Huber W, Anders S. Moderated estimation of fold change and dispersion for RNA-seq data with DESeq2. *Genome Biol* (2014) 15(12):550. doi: 10.1186/s13059-014-0550-8

21. Tibshirani R. The lasso method for variable selection in the Cox model. *Stat Med* (1997) 16(4):385-95. doi: 10.1002/(sici)1097-0258(19970228)16:4<385::aid-sim380>3.0.co;2-3

22. Engebretsen S, Bohlin J. Statistical predictions with glmnet. *Clin Epigenetics* (2019) 11(1):123. doi: 10.1186/s13148-019-0730-1

23. Zhang Q, Tan Y, Zhang J, Shi Y, Qi J, Zou D, et al. Pyroptosis-Related signature predicts prognosis and immunotherapy efficacy in Muscle-Invasive bladder cancer. *Front Immunol* (2022) 13(782982. doi: 10.3389/fimmu.2022.782982

24. Liu Z, Qi T, Li X, Yao Y, Othmane B, Chen J, et al. A novel TGF-beta risk score predicts the clinical outcomes and tumour microenvironment phenotypes in bladder cancer. *Front Immunol* (2021) 12(791924. doi: 10.3389/fimmu.2021.791924

25. Cao R, Ma B, Wang G, Xiong Y, Tian Y, Yuan L. Characterization of hypoxia response patterns identified prognosis and immunotherapy response in bladder cancer. *Mol Ther Oncolytics* (2021) 22(277-93. doi: 10.1016/j.omto.2021.06.011

26. Meng J, Lu X, Zhou Y, Zhang M, Ge Q, Zhou J, et al. Tumor immune microenvironment-based classifications of bladder cancer for enhancing the response rate of immunotherapy. *Mol Ther Oncolytics* (2021) 20(410-21. doi: 10.1016/j.omto.2021.02.001

27. Li H, Liu S, Li C, Xiao Z, Hu J, Zhao C. TNF Family-Based signature predicts prognosis, tumor microenvironment, and molecular subtypes in bladder carcinoma. *Front Cell Dev Biol* (2021) 9(800967. doi: 10.3389/fcell.2021.800967

28. Warrick JI, Sjodahl G, Kaag M, Raman JD, Merrill S, Shuman L, et al. Intratumoral heterogeneity of bladder cancer by molecular subtypes and histologic variants. *Eur Urol* (2019) 75(1):18-22. doi: 10.1016/j.eururo.2018.09.003

29. Kamoun A, de Reynies A, Allory Y, Sjodahl G, Robertson AG, Seiler R, et al. A consensus molecular classification of muscle-invasive bladder cancer. *Eur Urol* (2020) 77(4):420-33. doi: 10.1016/j.eururo.2019.09.006

30. Damrauer JS, Hoadley KA, Chism DD, Fan C, Tiganelli CJ, Wobker SE, et al. Intrinsic subtypes of high-grade bladder cancer reflect the hallmarks of breast cancer biology. *Proc Natl Acad Sci U S A* (2014) 111(8):3110-5. doi: 10.1073/pnas.1318376111

31. Robertson AG, Kim J, Al-Ahmadie H, Bellmunt J, Guo G, Cherniack AD, et al. Comprehensive molecular characterization of Muscle-Invasive bladder cancer. *Cell* (2017) 171(3):540-56. doi: 10.1016/j.cell.2017.09.007

32. Choi W, Porten S, Kim S, Willis D, Plimack ER, Hoffman-Censits J, et al. Identification of distinct basal and luminal subtypes of muscle-invasive bladder cancer with different sensitivities to frontline chemotherapy. *Cancer Cell* (2014) 25(2):152-65. doi: 10.1016/j.ccr.2014.01.009

33. Marzouka NA, Eriksson P, Rovira C, Liedberg F, Sjodahl G, Hoglund M. A validation and extended description of the Lund taxonomy for urothelial carcinoma using the TCGA cohort. *Sci Rep* (2018) 8(1):3737. doi: 10.1038/s41598-018-22126-x

34. Rebouissou S, Bernard-Pierrot I, de Reynies A, Lepage ML, Krucker C, Chapeaublanc E, et al. EGFR as a potential therapeutic target for a subset of muscle-invasive bladder cancers presenting a basal-like phenotype. *Sci Transl Med* (2014) 6(244):244r-291r. doi: 10.1126/scitranslmed.3008970

35. Kamoun A, de Reynies A, Allory Y, Sjodahl G, Robertson AG, Seiler R, et al. A consensus molecular classification of muscle-invasive bladder cancer. *Eur Urol* (2020) 77(4):420-33. doi: 10.1016/j.eururo.2019.09.006

36. Mo Q, Nikolos F, Chen F, Tramel Z, Lee YC, Hayashi K, et al. Prognostic power of a tumor differentiation gene signature for bladder urothelial carcinomas. *J Natl Cancer Inst* (2018) 110(5):448-59. doi: 10.1093/jnci/djx243

37. Mayakonda A, Lin DC, Assenov Y, Plass C, Koeffler HP. Maftools: Efficient and comprehensive analysis of somatic variants in cancer. *Genome Res* (2018) 28(11):1747-56. doi: 10.1101/gr.239244.118

38. Mermel CH, Schumacher SE, Hill B, Meyerson ML, Beroukhim R, Getz G. GISTIC2.0 facilitates sensitive and confident localization of the targets of focal somatic copy-number alteration in human cancers. *Genome Biol* (2011) 12(4):R41. doi: 10.1186/gb-2011-12-4-r41

39. Zhang H, Meltzer P, Davis S. RCircos: An R package for Circos 2D track plots. *Bmc Bioinformatics* (2013) 14(244. doi: 10.1186/1471-2105-14-244

40. Thorsson V, Gibbs DL, Brown SD, Wolf D, Bortone DS, Ou YT, et al. The immune landscape of cancer. *Immunity* (2018) 48(4):812-30. doi: 10.1016/j.immuni.2018.03.023

41. Cerami E, Gao J, Dogrusoz U, Gross BE, Sumer SO, Aksoy BA, et al. The cBio cancer genomics portal: An open platform for exploring multidimensional cancer genomics data. *Cancer Discov* (2012) 2(5):401-4. doi: 10.1158/2159-8290.CD-12-0095

42. Cedoz PL, Prunello M, Brennan K, Gevaert O. MethylMix 2.0: An R package for identifying DNA methylation genes. *Bioinformatics* (2018) 34(17):3044-6. doi: 10.1093/bioinformatics/bty156

43. Ryan M, Wong WC, Brown R, Akbani R, Su X, Broom B, et al. TCGASpliceSeq a compendium of alternative mRNA splicing in cancer. *Nucleic Acids Res* (2016) 44(D1):D1018-22. doi: 10.1093/nar/gkv1288

44. Li T, Fan J, Wang B, Traugh N, Chen Q, Liu JS, et al. TIMER: A web server for comprehensive analysis of Tumor-Infiltrating immune cells. *Cancer Res* (2017) 77(21):e108-10. doi: 10.1158/0008-5472.CAN-17-0307

45. Finotello F, Mayer C, Plattner C, Laschober G, Rieder D, Hackl H, et al. Molecular and pharmacological modulators of the tumor immune contexture revealed by deconvolution of RNA-seq data. *Genome Med* (2019) 11(1):34. doi: 10.1186/s13073-019-0638-6

46. Aran D, Hu Z, Butte AJ. XCell: Digitally portraying the tissue cellular heterogeneity landscape. *Genome Biol* (2017) 18(1):220. doi: 10.1186/s13059-017-1349-1

47. Chen B, Khodadoust MS, Liu CL, Newman AM, Alizadeh AA. Profiling tumor infiltrating immune cells with CIBERSORT. *Methods Mol Biol* (2018) 1711(243-59. doi: 10.1007/978-1-4939-7493-1_12

48. Becht E, Giraldo NA, Lacroix L, Buttard B, Elarouci N, Petitprez F, et al. Estimating the population abundance of tissue-infiltrating immune and stromal cell populations using gene expression. *Genome Biol* (2016) 17(1):218. doi: 10.1186/s13059-016-1070-5

49. Miao YR, Zhang Q, Lei Q, Luo M, Xie GY, Wang H, et al. ImmuCellAI: A unique method for comprehensive T-Cell subsets abundance prediction and its application in cancer immunotherapy. *Adv Sci (Weinh)* (2020) 7(7):1902880. doi: 10.1002/advs.201902880

50. Ru B, Wong CN, Tong Y, Zhong JY, Zhong S, Wu WC, et al. TISIDB: An integrated repository portal for tumor-immune system interactions. *Bioinformatics* (2019) 35(20):4200-2. doi: 10.1093/bioinformatics/btz210

51. Racle J, Gfeller D. EPIC: A tool to estimate the proportions of different cell types from bulk gene expression data. *Methods Mol Biol* (2020) 2120(233-48. doi: 10.1007/978-1-0716-0327-7_17

52. Yang W, Soares J, Greninger P, Edelman EJ, Lightfoot H, Forbes S, et al. Genomics of Drug Sensitivity in Cancer (GDSC): A resource for therapeutic biomarker discovery in cancer cells. *Nucleic Acids Res* (2013) 41(Database issue):D955-61. doi: 10.1093/nar/gks1111

53. Maeser D, Gruener RF, Huang RS. OncoPredict: An R package for predicting in vivo or cancer patient drug response and biomarkers from cell line screening data. *Brief Bioinform* (2021) 22(6). doi: 10.1093/bib/bbab260

54. Corsello SM, Nagari RT, Spangler RD, Rossen J, Kocak M, Bryan JG, et al. Discovering the anti-cancer potential of non-oncology drugs by systematic viability profiling. *Nat Cancer* (2020) 1(2):235-48. doi: 10.1038/s43018-019-0018-6

55. Seashore-Ludlow B, Rees MG, Cheah JH, Cokol M, Price EV, Coletti ME, et al. Harnessing connectivity in a Large-Scale Small-Molecule sensitivity dataset. *Cancer Discov* (2015) 5(11):1210-23. doi: 10.1158/2159-8290.CD-15-0235

56. Yang C, Huang X, Li Y, Chen J, Lv Y, Dai S. Prognosis and personalized treatment prediction in TP53-mutant hepatocellular carcinoma: An in silico strategy towards precision oncology. *Brief Bioinform* (2021) 22(3). doi: 10.1093/bib/bbaa164

57. Geeleher P, Cox N, Huang RS. PRRophetic: An R package for prediction of clinical chemotherapeutic response from tumor gene expression levels. *Plos One* (2014) 9(9):e107468. doi: 10.1371/journal.pone.0107468

58. Necchi A, Joseph RW, Loriot Y, Hoffman-Censits J, Perez-Gracia JL, Petrylak DP, et al. Atezolizumab in platinum-treated locally advanced or metastatic urothelial carcinoma: Post-progression outcomes from the phase II IMvigor210 study. *Ann Oncol* (2017) 28(12):3044-50. doi: 10.1093/annonc/mdx518

59. Langfelder P, Horvath S. WGCNA: An R package for weighted correlation network analysis. *Bmc Bioinformatics* (2008) 9(559. doi: 10.1186/1471-2105-9-559

60. Shen W, Song Z, Zhong X, Huang M, Shen D, Gao P, et al. Sangerbox: A comprehensive, interaction‐friendly clinical bioinformatics analysis platform. *iMeta* (2022). doi: 10.1002/imt2.36
