# Supplementary figures and images for: Identification of molecular subtypes based on liquid–liquid phase separation and cross-talk with immunological phenotype in bladder cancer
Source: Front Immunol. 2022 Nov 28;13:1059568. doi: 10.3389/fimmu.2022.1059568 (PMC9742536; doi:10.3389/fimmu.2022.1059568)

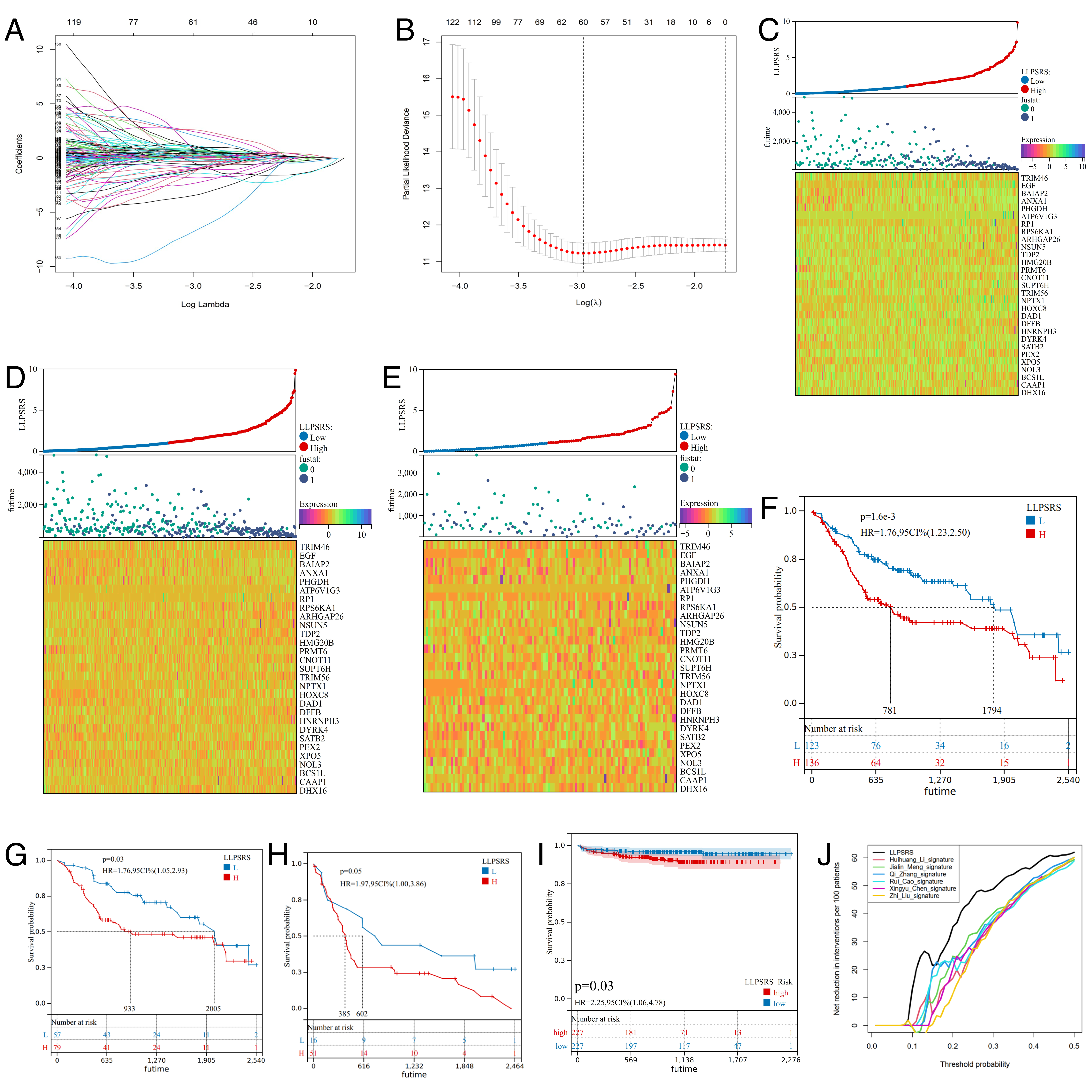

Supplement: Supplementary Figure 3 — (A, B) The relation between lambda values and variable coefficients or partial likelihood deviance in the LASSO regression. (C–E) Patients were divided into high- and low-risk subgroup in the training set, TCGA-BLCA cohort, and validation set; survival status of patients in two subgroups; heatmap of 29 RSGs. (F–I) KM curves for LLPSRS in the external validation sets (Meta-BLCA, GSE13507, GSE31684, E-MTAB-4321 cohorts). (I–N) Comparison of LLPSRS with other six prognostic signatures in terms of DCA, C-index and ROC-AUC values at 1, 3, 5 and 7 years. [file Image_3.tif]

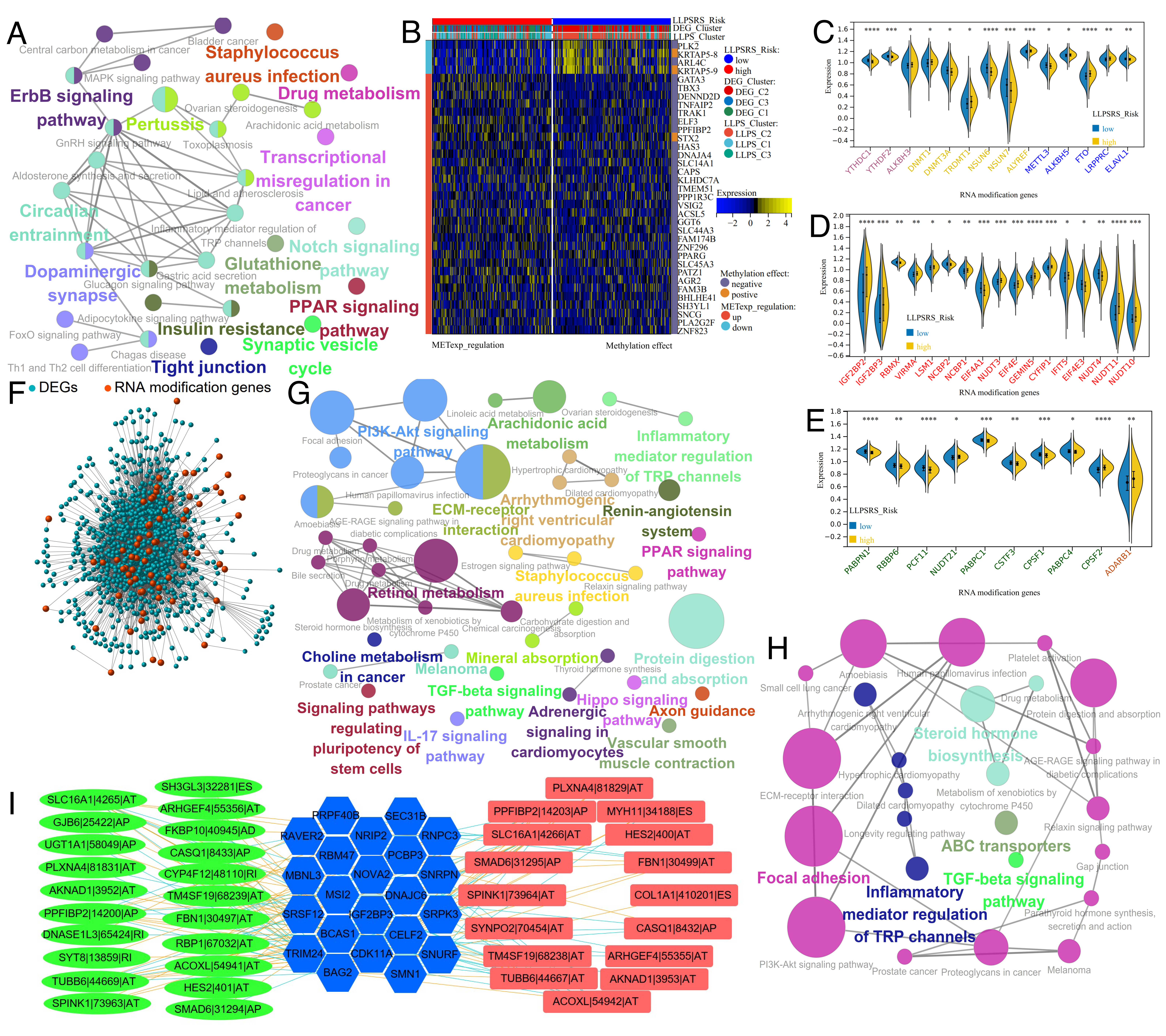

Supplement: Supplementary Figure 5 — (A) KEGG annotation of 240 DNA methylation-relevant driver genes. The different colors represented the different terms or pathways. (B) Heatmap showed the DNA methylation levels of top 35 hypermethylated or hypomethylated genes. (C–E) Differential expression of RNA modification regulators between high- and low- risk subgroups. Color code of the regulators indicated corresponding modification type. (F) Network diagram showed that 786 of DEGs were discovered to have co-expression relations with 71 RNA editing regulators with |Pearson’s r| > 0.3 and p< 0.0001 as the threshold. (G) KEGG annotation of 786 RNA editing-relevant DEGs. The different colors represented the different terms or pathways. (H) KEGG annotation of 161 DEGs with AS. The different colors represented the different terms or pathways. (I) The splicing regulatory network between the prognostic AS events of DEGs and relevant splicing factors. [file Image_5.tif]
